# The Neural Correlates of Individual Differences in Reinforcement Learning during Pain Avoidance and Reward Seeking

**DOI:** 10.1523/ENEURO.0437-23.2024

**Published:** 2024-02-23

**Authors:** Thang M. Le, Takeyuki Oba, Luke Couch, Lauren McInerney, Chiang-Shan R. Li

**Affiliations:** ^1^Department of Psychiatry, Yale University School of Medicine, New Haven, Connecticut 06519; ^2^Human Informatics and Interaction Research Institute, the National Institute of Advanced Industrial Science and Technology (AIST), Tsukuba 305-8560, Japan; ^3^Department of Neuroscience, Yale University School of Medicine, New Haven, Connecticut 06520; ^4^Interdepartmental Neuroscience Program, Yale University School of Medicine, New Haven, Connecticut 06520; ^5^Wu Tsai Institute, Yale University, New Haven, Connecticut 06510

**Keywords:** anterior cingulate cortex, avoidance learning, fMRI, pain, reinforcement learning, reward

## Abstract

Organisms learn to gain reward and avoid punishment through action–outcome associations. Reinforcement learning (RL) offers a critical framework to understand individual differences in this associative learning by assessing learning rate, action bias, pavlovian factor (i.e., the extent to which action values are influenced by stimulus values), and subjective impact of outcomes (i.e., motivation to seek reward and avoid punishment). Nevertheless, how these individual-level metrics are represented in the brain remains unclear. The current study leveraged fMRI in healthy humans and a probabilistic learning go/no-go task to characterize the neural correlates involved in learning to seek reward and avoid pain. Behaviorally, participants showed a higher learning rate during pain avoidance relative to reward seeking. Additionally, the subjective impact of outcomes was greater for reward trials and associated with lower response randomness. Our imaging findings showed that individual differences in learning rate and performance accuracy during avoidance learning were positively associated with activities of the dorsal anterior cingulate cortex, midcingulate cortex, and postcentral gyrus. In contrast, the pavlovian factor was represented in the precentral gyrus and superior frontal gyrus (SFG) during pain avoidance and reward seeking, respectively. Individual variation of the subjective impact of outcomes was positively predicted by activation of the left posterior cingulate cortex. Finally, action bias was represented by the supplementary motor area (SMA) and pre-SMA whereas the SFG played a role in restraining this action tendency. Together, these findings highlight for the first time the neural substrates of individual differences in the computational processes during RL.

## Significant Statement

Learning how to gain reward and avoid punishment is critical for survival. Reinforcement learning models offer several measures characterizing such learning including learning rate, action bias, pavlovian factor, and subjective impact of outcomes. Yet, the brain substrates subserving individual differences in these metrics remain unclear. The current study identified the distinct involvement of the anterior, mid-, and posterior cingulate cortex, along with the SMA and superior frontal gyrus in representing distinct learning metrics that influence how individuals learn to initiate or inhibit an action to gain reward and avoid painful outcomes. Our findings help delineate the neural processes that may shed light on action choices, future behavior prediction, and the pathology of mental illnesses that implicate learning dysfunctions.

## Introduction

A fundamental inquiry in behavioral neuroscience concerns the characterization of the brain processes underlying one's choice of action to maximize rewards and minimize punishment. Such decision-making processes can be understood using reinforcement learning (RL) theories (Sutton and Barto, 1998). In RL, an agent learns the values of choices by forming associations between actions and outcomes. These associations help predict the expected values for a particular set of actions and then bias action selection toward the agent's goals (O’Doherty et al., 2015). With roots in computer science and neurophysiological research, RL models offer a conceptual framework to identify distinct component processes of learning (Hampton et al., 2006; Niv, 2009). Specifically, RL models generate several parameters capturing learning rate, action bias, and subjective impact of outcomes that characterize differences in learning across individuals. It is suggested that the brain areas involved in goal-directed behaviors likely harbor signals related to these component variables (Lee et al., 2012). Describing such signals would be of critical importance in elucidating the neurobiology of decision-making, predicting future behaviors, and understanding the pathology of mental illnesses that implicate dysfunctional avoidance and/or reward learning.

A number of studies have investigated the neural correlates of RL model-derived indices of associative learning. For instance, Guitart-Masip et al. (2012) used a probabilistic learning go/no-go task (PLGT) with monetary gain and loss to localize the effects of action values in the striatum and substantia nigra/ventral tegmental area. Employing a social reinforcement task, another study reported higher pregenual anterior cingulate cortex (ACC) activation with increased expected stimulus values (Jones et al., 2011). The ventromedial prefrontal cortex was also found to track learned values of options in an RL task (Jocham et al., 2011). These investigations highlighted the brain underpinnings of model variables in trial-by-trial variation that are shared across participants, with an emphasis on reward learning. Despite the increasing interest in the neural bases of RL, the neural correlates of individual differences in RL model parameters remain underexplored.

RL models offer several indices that describe learning. For instance, learning rate assesses the change in action following an error, a metric that quantifies one's adaptive behavior (Oba et al., 2019). The subjective impact of outcomes which indicates the motivation to seek reward or avoid punishment is critical to individual differences in goal-directed behaviors. pavlovian factor, referring to the extent to which action values are influenced by stimulus values, independent of learning, determines the extent to which actions are motivated by outcomes. Another useful measure is action bias which assesses an individual's tendency to initiate an action regardless of the optimal strategy. Importantly, these individual differences at the extreme may suggest psychopathology in mental illnesses that involve learning dysfunctions. For instance, reward-driven impulsivity is widely implicated in substance use disorders and behavioral addiction (Jentsch et al., 2014; Maxwell et al., 2020). In contrast, while learning to avoid aversive outcomes such as pain is critical to the survival and well-being of organisms, avoidance learning dysfunctions represent a detrimental feature in anxiety, obsessive-compulsive, posttraumatic stress, and substance use disorders (Radell et al., 2020). Thus, characterizing the individual differences both during reward and punishment learning will inform research on the pathophysiology of mental disorders.

Ample research has examined neuronal responses and regional activation to motivated actions. In particular, studies examining reward learning have implicated the ACC in representing learned cue values (Jones et al., 2011), past reward weights (Wittmann et al., 2016), and reinforcement history (Holroyd and Coles, 2008). The dorsal ACC, in particular, has been found to exhibit significantly higher activation during reward learning in learners versus nonlearners (Santesso et al., 2008). In contrast, the posterior cingulate cortex (PCC) has been implicated in actions aimed at avoiding punishment both in humans (Roy et al., 2011; Le et al., 2019) and animals (Riekkinen et al., 1995; Duvel et al., 2001), suggesting a role in avoidance learning. It is likely that the ACC and PCC circuits are involved in representing individual differences in reward and punishment learning.

Previous RL research has largely associated reward and punishment with approach and avoidance behaviors, respectively. However, punishment avoidance may at times require the initiation rather than suppression of an action to avert a negative event. Similarly, reward seeking may necessitate response inhibition for a successful outcome, likely engaging distinct regional activities (Groman et al., 2022). Thus, to investigate the neural correlates of RL model parameters, we employed a PLGT that fully orthogonalized action types and motivational goals in a balanced design (go vs no-go) × (pain vs reward). We used a whole-brain approach to identify the neural correlates of RL model parameters, including learning rate, subjective impact of outcomes, action bias, and pavlovian factor. We hypothesized that the ACC and PCC would demonstrate regional responses that reflect individual variation in the parameters of the RL model that best characterizes behavioral performance.

## Materials and Methods

### Participants

Eighty-two healthy adults (34 women, 35.9 ± 11.2 years in age) participated in the study. All participants were screened to be free from major medical, neurological, and Axis I psychiatric disorders. No participants were currently on psychotropic medications, and all tested negative for illicit substances on the study day. Subjects provided written informed consent after details of the study were explained, in accordance with the institute guidelines and procedures approved by the Institutional Human Investigation Committee.

### PLGT

**Figure 1.**
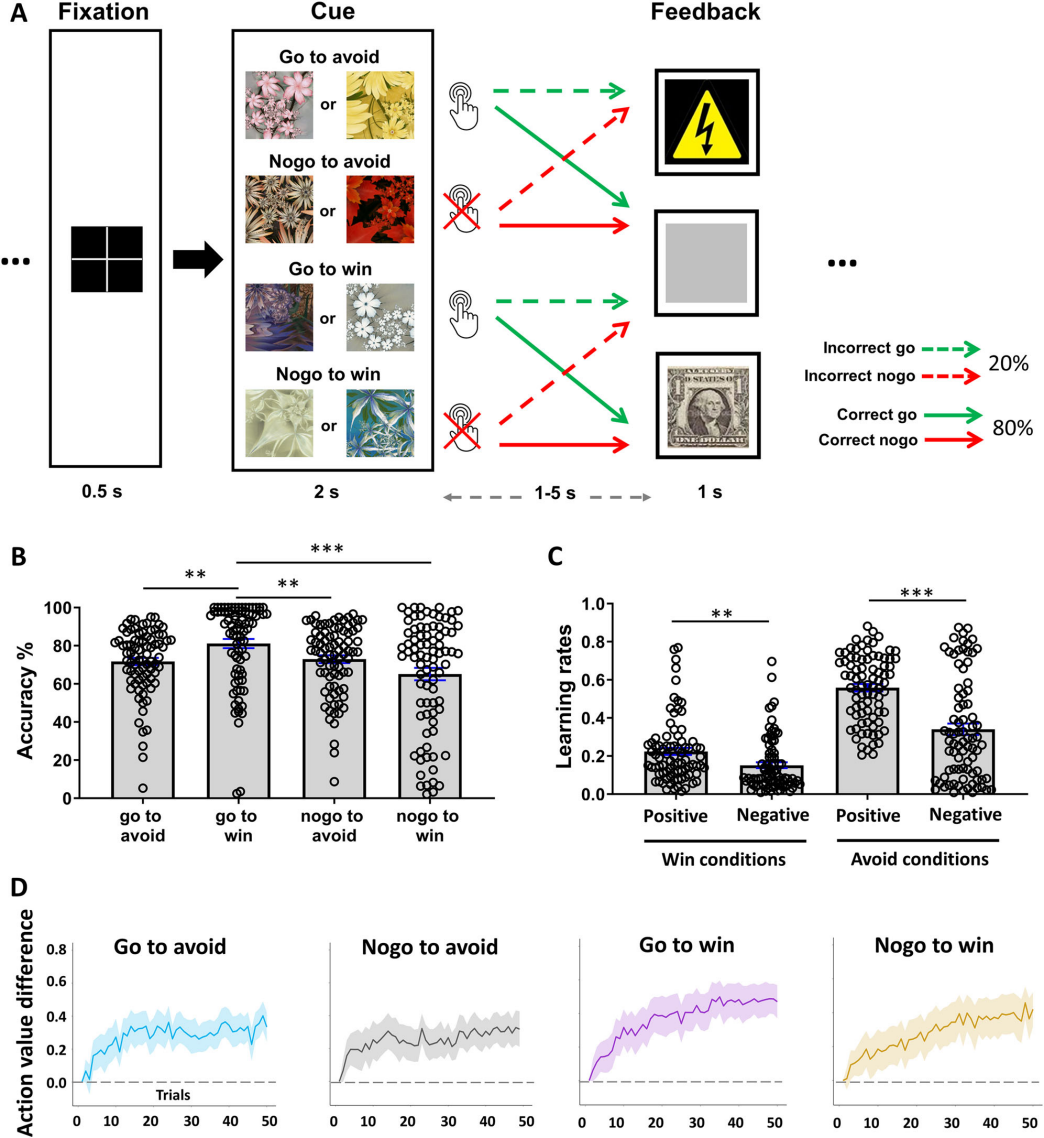
***A***, PLGT: participants learned to respond to four cue categories to avoid electric shocks (i.e., go-to-avoid, no-go-to-avoid) and gain monetary rewards (i.e., go-to-win, no-go-to-win), with two different images per cue category. Correct responses yielded favorable outcomes 80% of the time whereas incorrect responses yielded unfavorable outcomes 80% of the time. Shocks were only delivered in 50% of the shock feedback instances to reduce head movement. ***B***, Performance accuracy in the four conditions: participants performed significantly better in go-to-win than in any other conditions. **p ≤ 0.01; ***p ≤ 0.001. ***C***, Learning rates: in monetary win and pain avoidance conditions, learning was separately estimated when unexpected favorable outcomes (positive) or unexpected unfavorable outcomes (negative) were encountered. ***D***, Action value difference: subjects showed learning of the two go cues (go-to-avoid and go-to-win), as demonstrated by the increasing difference between go and no-go action values over time. A similar pattern of learning was found for the no-go trials as the difference between no-go and go action values also exhibited an upward trend as the trials progressed. Shade indicates confidence interval.

Participants underwent fMRI while performing the PLGT (Fig. 1*A*; Guitart-Masip et al., 2012; Oba et al., 2019). In each run, a cue (fractal) image was presented at the beginning of a trial to signal one of the four contingencies: go to win $1, no-go to win $1, go to avoid a painful shock, or no-go to avoid shock. There were eight images, two per cue category, with cue–outcome mappings randomized across participants. The cue was displayed for 2 s, and participants were instructed to decide whether to press a button (go) or not (no-go) before it disappeared. After a randomized interval of 1–5 s, feedback of reward (win trials), shock (avoid trials), or “null” (both win and avoid trials) was delivered. The intertrial interval varied randomly from 2 to 8 s. The randomization and intervening time intervals enabled the modeling of distinct regional responses to anticipation and feedback. The outcome was probabilistic, with 80%/20% of correct/incorrect responses in the win trials rewarded and the remaining 20%/80% of correct/incorrect responses leading to a null outcome (Fig. 1*A*). In avoid trials, electric shocks were avoided (a null outcome) on 80%/20% of correct/incorrect responses, with the remainder leading to electric shocks. In reality, despite the feedback display of the shock image, shocks were randomly delivered only half of the time to minimize head movements. Each shock was followed by a 20 s rest window to allow neural and physiological responses to return to baseline.

Prior to MRI, an appropriate pain intensity level was calibrated for each participant so that the shocks would be painful but tolerable. Participants also practiced on a variant of the task with identical rules, with the exception that there was one image per cue category.

With four 10 min learning runs, there were approximately 50 trials for each cue. Participants won ∼$78 on average (plus a base payment of $25) and experienced a total of 14 actual shocks and 14 omitted shocks on average.

### RL models

We constructed RL models of the subjects’ behavioral data. A detailed description of the models can be found in our previous work (Oba et al., 2019) and elsewhere (Guitart-Masip et al., 2012). All models assigned an action value to each action in a given trial. For instance, considering action *a* (go or no-go) in response to cue *s* on trial *t*, the action value *Q_t_*(*a_t_*, *s_t_*) for a chosen action was updated based on the following equation:

(1) $$Q_{t+1}(a_{t},s_{t})=Q_{t}(a_{t},s_{t})+\varepsilon \delta_{t},$$

(2) $$\delta_{t}=\rho r_{t}-Q_{t}(a_{t},s_{t}),$$

where *ε* was the learning rate governing how the action value was updated. The subjective impact of outcome *ρ* was a free parameter representing the effect size of reinforcement for a subject. The outcome value *r_t_* was +1 for a gain, −1 for a shock, or 0 for no gain or shock in trial *t*. The term *ρr_t_* −*Q_t_*(*a_t_*, *s_t_*) represented the prediction error (PE) or *δ_t_*. Learning proceeded with a decision for each action according to the values, and the probabilities of implementing an action were calculated by the softmax function:

(3) $$p_{t}(a_{t},s_{t})=\frac{\exp(W_{t}(a_{t},s_{t}))}{\sum_{a^{\prime }}\exp(W_{t}({a^{\prime }}_{t},s_{t}))},$$

where *W_t_*(*a_t_*, *s_t_*) was an action weight corresponding to *Q_t_*(*a_t_*, *s_t_*).

In addition, we included two other parameters validated in prior studies to better explain behavioral performance (Guitart-Masip et al., 2012, 2014). One parameter was the action bias *b*, a tendency to press a button regardless of learning, which influenced the action value on the weight:

(4) $$W_{t}(a_{t},s_{t})=\{\begin{array}{c} Q_{t}(a_{t},\;s_{t})+b\;\;\mathrm{if}\;a_{t}=\mathrm{go}\\ Q_{t}(a_{t},\;s_{t})\;\;\mathrm{else} \end{array}.$$

The other parameter was the pavlovian factor, which expresses the effect of a stimulus value independent of learning. Several studies have reported that stimuli resulting in rewards tend to block action inhibition, thus reflecting an approach bias (Guitart-Masip et al., 2012, 2014). The action weight is adapted by the pavlovian factor *π* as follows:

(5) $$W_{t}(a_{t},s_{t})\;=\{\begin{array}{c} Q_{t}(a_{t},\;s_{t})+\pi V_{t}(s_{t})\;\;\mathrm{if}\;a_{t}=\mathrm{go}\\ Q_{t}(a_{t},\;s_{t})\;\;\mathrm{else} \end{array},$$

(6) $$V_{t+1}(s_{t})=V_{t}+\varepsilon (\rho r_{t}\text{–}V_{t}(s_{t})).$$

The stimulus value *V_t_*(*s_t_*) was updated with the same parameters as the action value. The outputs of the models included the learning rate *ε*, which can be separated according to the sign of PE (positive, *δ* > 0, or negative, *δ* < 0). Models that produce the learning rates for the signed PE allow asymmetric effects of better or worse (than expected) outcomes on learning (Cazé and van der Meer, 2013). Furthermore, the learning rates can be modeled separately for avoid and reward trials; thus, the models would have four different learning rates: *ε*WP for win trials with a positive PE, *ε*WN for win trials with a negative PE, *ε*AP for avoid trials with a positive PE, and *ε*AN for avoid trials with a negative PE. The subjective impact of outcomes could also differ between win (*ρ*W) and avoid (*ρ*A) trials, as the subjective impacts of positive and negative reinforcers may not be the same. In sum, we examined a total of 12 parameters and identified the best combination of these parameters in modeling the behavioral data.

Free parameters were estimated for each participant via a hierarchical type II maximum likelihood procedure, as with previous studies (Huys et al., 2011; Guitart-Masip et al., 2012). We supposed that the population-level distribution for each parameter was a normal distribution. To perform the estimation, the likelihood was maximized by the expectation–maximization procedure using the Laplace approximation to calculate the posterior probability. We used the Rsolnp package in R to optimize the likelihood functions. These models were evaluated with the integrated Bayesian information criterion (iBIC). A smaller iBIC value represents a better model (Huys et al., 2011). Briefly, the iBIC was calculated by using the following procedures: using the parameter values randomly generated by the population distributions, the likelihood was calculated 1,000 times for each participant data. Next, after dividing the total likelihood of each participant by the number of samples (1,000), these amounts were summed for all participants. Finally, the cost for the number of parameters was added to this value. Thus, the iBIC values approximated the log marginal likelihoods with a penalty for the number of free parameters. RL code was run on Lenovo Workstation TS P720, using the operating system Ubuntu 20.04.4 LTS.

### Imaging protocol and data preprocessing

Conventional T1-weighted spin-echo sagittal anatomical images were acquired for slice localization using a 3T scanner (Siemens Trio). Anatomical images of the functional slice locations were next obtained with spin-echo imaging in the axial plane parallel to the AC–PC line with (TR) = 1,900 ms, echo time (TE) = 2.52 ms, bandwidth = 170 Hz/pixel, FOV = 250 × 250 mm, matrix = 256 × 256, 176 slices with slice thickness = 1 mm, and no gap. Functional BOLD signals were acquired using multiband imaging (multiband acceleration factor = 3) with a single-shot gradient-echo echoplanar imaging sequence. Fifty-one axial slices parallel to the AC–PC line covering the whole brain were acquired with TR = 1,000 ms, TE = 30 ms, bandwidth = 2,290 Hz/pixel, flip angle = 62°, field of view = 210 × 210 mm, matrix = 84 × 84, slice thickness = 2.5 mm, and no gap.

Imaging data were preprocessed using SPM12 (Wellcome Trust Centre for Neuroimaging). Subjects with BOLD runs with significant motion (>3 mm translation peak-to-peak movement and/or 1.5° rotation) were removed. Furthermore, we calculated framewise displacement (FD) for each task run and removed subjects with an average FD of >0.2. This resulted in the removal of five subjects, leaving a sample of 82 subjects as reported above. Images from the first five TRs at the beginning of each run were discarded to ensure only BOLD signals at steady-state equilibrium between RF pulsing and relaxation were included in analyses. Physiological signals including respiration and heart rate were regressed out to minimize the influence of these sources of noise. Images of each subject were first realigned (motion corrected) and corrected for slice timing. A mean functional image volume was constructed for each subject per run from the realigned image volumes. These mean images were coregistered with the high-resolution structural image and then segmented for normalization with affine registration followed by nonlinear transformation. The normalization parameters determined for the structure volume were then applied to the corresponding functional image volumes for each subject. Images were resampled to 2.5 mm isotropic voxel size. Finally, the images were smoothed with a Gaussian kernel of 4 mm FWHM.

### Imaging data modeling and group analyses

We constructed a GLM to examine the shared and distinct brain processes underlying the initiation and inhibition of an action to either avoid pain or gain reward. To this end, we included two trial types, with cue onsets of individual trials convolved with a canonical HRF and with the temporal derivative of the canonical HRF and entered as regressors in the GLM. The two trial types included avoid and win trials, with go and no-go responses collapsed. This resulted in the contrasts avoid > 0 and win > 0. Motion regressors were included in the GLMs. We corrected for serial autocorrelation caused by aliased cardiovascular and respiratory effects by the FAST model. Finally, to examine potential action bias in subjects’ learning, we constructed the contrast go versus no-go, collapsing avoid and win trials.

To identify the neural correlates of RL model parameters, we used whole-brain multiple regressions at the group level with the following parameters each as the predictor of the brain activity during the cue period: learning rates (i.e., the magnitude of change of action following errors), pavlovian factor (i.e., the extent to which action values are influenced by stimulus values and not by learning), subjective impact of outcomes (i.e., motivation to seek reward and avoid punishment), and action bias (i.e., the tendency to initiate an action). For learning rate, pavlovian factor, and subjective impact of outcomes, we combined go and no-go trials. Furthermore, we averaged the two learning rates from positive outcomes and negative outcomes each for win trials (i.e., *ε*WP and *ε*WN) and avoid trials (i.e., *ε*AP and *ε*AN). This resulted in two learning rates, one for win trials and one for avoid trials. These parameters were used to predict regional responses to pain avoidance and reward seeking during the cue period (i.e., contrasts avoid > 0 and win > 0, respectively). For action bias, we used the contrasts go > no-go and no-go > go to predict regional responses to action initiation and inhibition, respectively. All multiple regressions controlled for sex and age. As the total number of shocks showed a significant relationship with the pavlovian factor (*r* = 0.30, *p* = 0.007), the subjective impact of outcomes during avoidance (*r* = −0.48, *p* < 0.001), and action bias (*r* = 0.22, *p* = 0.04), we also included this variable as a covariate in the multiple regressions involving avoidance learning.

The results of all whole-brain analyses were evaluated with voxel *p* < 0.001 in combination with cluster *p* < 0.05, corrected for family-wise error of multiple comparisons, according to current reporting standards (Woo et al., 2014; Eklund et al., 2016). Minimum cluster size was 30. All peaks of activation were reported in MNI coordinates.

### Stay/switch analysis

In addition to accuracy performance and response time, we conducted a stay/switch analysis to evaluate the subjects’ task performance. The analysis yielded the percentage of instances in which a subject immediately switched their response (i.e., from go to no-go or no-go to go) after encountering an unfavorable outcome (e.g., null feedback in a win trial or shock feedback in an avoid trial). Switching was only advantageous if the previous response consistently yielded unfavorable outcomes. Due to the probabilistic nature of our task, excessive switching likely indicated poor learning. Previous studies showed that increased switching was associated with the lack of a coherent task strategy and “reward chasing” in individuals with substance use disorders (Myers et al., 2016; Robinson et al., 2021).

### Mediation analysis

We conducted mediation analyses using a single-mediator model to examine the interrelationship between regional activities, learning parameters estimated by the model, and performance variables. The methods are detailed in a previous work (MacKinnon et al., 2007). Briefly, in a mediation analysis, the relation between the independent variable *X* and the dependent variable *Y*; that is, *X* → *Y* is tested to determine whether it is significantly mediated by a variable *M*. The mediation test is performed using the following three regression equations:

Y=i1+cX+e1,

$$Y=i2+c^{\prime }X+bM+e2,$$

M=i3+aX+e3,

where *a* represents *X* → *M*, *b* represents *M* → *Y* (controlling for *X*), *c*′ represents *X* → *Y* (controlling for *M*), and *c* represents *X* → *Y*. *a*, *b*, *c*, and *c*′ are referred to as “path coefficients” or simply “paths.” Variable *M* is said to be a mediator of connection *X* → *Y*, if (*c* – *c*′), which is mathematically equivalent to the product of the paths *a* × *b*, is significantly different from zero (MacKinnon et al., 2007). If (*c* – *c*′) is different from zero and the paths *a* and *b* are significant, one concludes that *X* → *Y* is mediated by *M*. In addition, if path *c*′ is not significant, there is no direct connection from *X* to *Y*, and thus *X* → *Y* is completely mediated by *M*. Note that path *b* represents *M* → *Y*, controlling for *X*, and should not be confused with the correlation coefficient between *Y* and *M*. Significant correlations between *X* and *Y* and between *X* and *M* are required for one to perform the mediation test. The analysis was performed with package Lavaan (Rosseel, 2012) in R (https://www.r-project.org). To test the significance of the mediation effect, we used the bootstrapping method (Preacher and Hayes, 2004) as it is generally considered advantageous to the Sobel test (MacKinnon et al., 2007).

Specifically, we evaluated the relationships of each of all four variables indexing learning (i.e., learning rate, pavlovian factor, subjective impact of outcomes, and action bias) with brain activation and task performance (see Results). For brain activations, we extracted the parameter estimates from the regions identified by the whole-brain multiple regressions with the learning parameters as the predictors of the contrast avoid > 0, win > 0, go > no-go, no-go > go for avoidance learning, reward learning, action initiation, and action inhibition, respectively. We tested the hypothesis that the relationship between brain activations and task performance was significantly mediated by the RL model parameters.

#### Code accessibility

The code/software described in the paper is freely available online at https://github.com/tml44/RL_models_PLGT/. The code is available as Extended Data.

10.1523/ENEURO.0154-22.2023.d1 Data 1 Code for the construction and evaluation of all RL models as described in the Methods. Fourteen models were constructed, producing 14 sets of parameters and iBIC. The optimal model included four different learning rates (εWP, εWN, εAP, and εAN) and two subjective impact of outcomes (ρW and <A), action bias b, and the Pavlovian factor π. Abbreviations: Ep: Learning rate, Rh: subjective impact of outcomes, Bi: Action bias, Pav: Pavlovian factor. Model inputs require behavioral data obtained from PLGT performance. Download Data 1, DOCX file.

## Results

### Behavioral results

We conducted a 2 (motivational goal, avoid vs win) × 2 (response type, go vs no-go) repeated-measures ANOVA of performance accuracy (Fig. 1*B*). The results showed no significant main effect of motivational goal (*p* = 0.06) or response type (*p* = 0.41). However, there was a significant interaction effect (*F*_(1,80)_ = 9.26, *p* = 0.002). Post hoc analyses revealed that performance accuracy was significantly higher for go-to-win conditions than no-go-to-win (*p* < 0.001), go-to-avoid (*p* = 0.002), and no-go-to-avoid (*p* = 0.01) conditions. As expected, avoid and win trials did not significantly differ in performance accuracy (*p* = 0.59). No other comparisons were significant.

During probabilistic learning, excessive switching (i.e., switching from go to no-go or vice versa for the same cue after one instance of unfavorable outcome) may indicate poor learning. Thus, we computed for each task condition a stay/switch index, which measures the percentage of switches immediately after an encounter with unfavorable feedback. Next, we performed a 2 (motivational goal, avoid vs win) × 2 (response type, go vs no-go) repeated-measures ANOVA. There was a significant main effect of motivational goal (*F*_(1,81)_ = 105.69, *p* < 0.001) but no significant main effect of response type (*p* = 0.49). The interaction effect was significant (*F*_(1,80)_ = 4.97, *p* = 0.02). Post hoc analyses showed that participants switched significantly more in avoid relative to win trials (*p* < 0.001) whereas there was no significant difference between go and no-go trials (*p* = 0.36). Next, we examined the relationship between the stay/switch index and task performance and found a significant negative correlation between the index and performance accuracy of avoid trials (*r* = −0.31, *p* = 0.005). The relationship was not significant for the win trials (*p* = 0.41). Taken together, participants displayed more inconsistent response behavior in avoid trials and such inconsistency negatively impacted task performance.

### RL model of performance

We constructed 14 models with different combinations of free parameters to determine the model that optimally predicted the choice data. Using a stepwise procedure for model comparison and selection, we added one free parameter to a model, calculated the iBIC, and accepted the parameter that decreased the iBIC the most at each step. The pavlovian factor *π* reduced the iBIC of the basic model (one learning rate *ε* and one subjective impact of outcomes *ρ*) over the other parameters. The iBIC of the model with *π* was diminished by the separation of the learning rates into positive and negative PEs (*ε*P and *ε*N). The iBIC value decreased further with learning rates computed separately for win (i.e., monetary win) and avoid (i.e., pain avoidance) trials (*ε*WP, *ε*WN, *ε*AP, and *ε*AN). The subjective impact of outcomes was separated for win (*ρ*W) and avoid (*ρ*A) trials. Finally, the action bias parameter *b* reduced the iBIC. Thus, the optimal model included four different learning rates (*ε*WP, *ε*WN, *ε*AP, and *ε*AN) and two subjective impact of outcomes (*ρ*W and *ρ*A), action bias *b*, and the pavlovian factor *π*.

With the four learning rates (*ε*WP, *ε*WN, *ε*AP, and *ε*AN; Fig. 1*C*), a 2 (condition, win vs avoid) × 2 (direction, positive vs negative PE) ANOVA showed a significant main effect of condition (*F*_(1,81)_ = 146.0, *p* < 0.001) and of learning direction (*F*_(1,81)_ = 45.02, *p* < 0.001), as well as a significant interaction effect (*F*_(1,80)_ = 11.45, *p* < 0.001). Post hoc comparisons revealed that subjects showed greater change in response following an error in avoiding shocks relative to winning money (*p* < 0.001) and in encountering favorable relative to unfavorable outcomes (*p*'s < 0.01).

We extracted the subjective impact of outcomes for win (*ρ*W) and avoid (*ρ*A) trials and found that *ρ*W was significantly greater than *ρ*A, suggesting that stimulus value was perceived to be greater for win trials. Interestingly, the stay/switch index for avoid and win trials was significantly correlated with *ρ*A (*r* = −0.31, *p* = 0.005) and *ρ*W (*r* = −0.30, *p* = 0.007), respectively. This finding indicates that the less value the subjects placed on the outcomes, the more likely the subjects showed inconsistent patterns of responses. In other words, the subjects with lower subjective impact of outcomes appeared to be more uncertain about the best action to take.

To further characterize subjects’ learning, we estimated for individual subjects the action values *Q_t_*(*go*) and *Q_t_*(*no-go*) and computed the difference *Q_t_*(*go*) versus *Q_t_*(*no-go*) for each trial. We expected that, for go trials, learning would manifest as the increase of *Q_t_*(*go*)–*Q_t_*(*no-go*) over time. In contrast, for no-go trials, *Q_t_*(*no-go*)–*Q_t_*(*go*) would rise as the trials progressed. The results were consistent with our prediction (Fig. 1*D*). In other words, subjects placed increasingly more value on go versus no-go action as they encountered more go trials, whereas the reverse was true for no-go trials. These findings suggest subjects showed learning across all four trial types over time.

### Imaging results

#### Neural correlates of individual differences in learning rates

**Figure 2.**
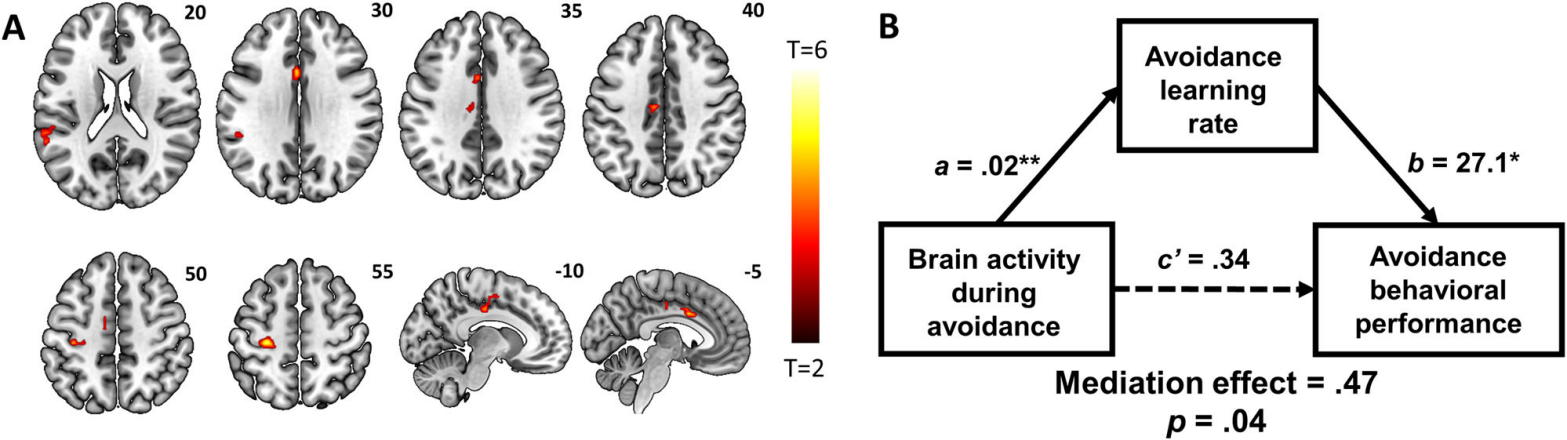
Neural correlates of avoidance learning rate during pain avoidance. ***A***, Whole-brain multiple regression of avoid > 0 on learning rate showed higher activations in the dorsal ACC, MCC, left PoCG, and left superior temporal sulcus. ***B***, The regional activations positively predicted performance accuracy, and this relationship was significantly mediated by the learning rate during pain avoidance. *p < 0.05; **p < 0.01.

To identify the neural correlates of learning, we conducted whole-brain multiple regressions, first with the learning rate during pain avoidance. A small *ε* indicated that the individual was less inclined to modify their future responses following the outcomes. The results showed that, with go and no-go trials collapsed, *ε* predicted activations to avoid > 0 during the cue period in the dACC, midcingulate cortex (MCC), left postcentral gyrus (PoCG), and left superior temporal sulcus (Fig. 2*A*, Table 1). No clusters showed a significant negative relationship.

**Table 1.** Neural correlates of individual differences in RL parameters during pain avoidance and reward seeking

Region	MNI coordinates (mm)			Voxel *T*	Cluster *k*
	*x*	*y*	*z*		
Learning rate during Avoid > 0					
Dorsal ACC	−2	9	33	5.30	54
PoCG	−28	−26	53	5.54	80
	−35	−26	48	4.55	
	−28	−21	68	4.29	
Superior temporal sulcus	−60	−48	26	4.85	82
	−52	−41	28	4.54	
MCC	−8	−16	38	4.56	47
	−10	−16	38	3.69	
Pavlovian factor during avoid < 0					
PrCG	−45	−11	40	4.29	33
	−52	−16	46	4.04	
Pavlovian factor during win < 0					
SFG	25	44	26	5.24	35
PCC	10	−56	26	4.96	45
	5	−61	33	4.71	
Subjective impact of outcomes during avoid > 0					
SPL	−42	−51	40	4.41	34
	−42	−51	28	3.97	
PCC	2	−44	30	4.23	66
	8	−48	18	3.80	
	8	−54	40	3.72	

Notably, the learning rate and performance accuracy were positively correlated during pain avoidance (*r* = 0.31, *p* = 0.005). To remove the effects of sex, age, and number of total shocks in this correlation, as is the case with all following correlations, we regressed out these variables (i.e., partial correlation). The activities (i.e., parameter estimates, or *β*'s) of brain regions identified by the whole-brain multiple regression were also significantly correlated with performance accuracy (*r* = 0.36, *p* = 0.001). Using the mediation analysis, we determined a significant model in which the relationship between the brain activity during pain avoidance and performance accuracy was significantly and fully mediated by learning rate (Fig. 2*B*). Thus, the greater the activations in the dACC, MCC, left PoCG, and left superior temporal sulcus, the better the performance accuracy; and this relationship was positively and fully mediated by learning rate during pain avoidance.

The multiple regression applied to the learning rate of reward seeking (i.e., win > 0) did not yield any significant results at the same threshold.

#### Neural correlates of individual differences in pavlovian factor

**Figure 3.**
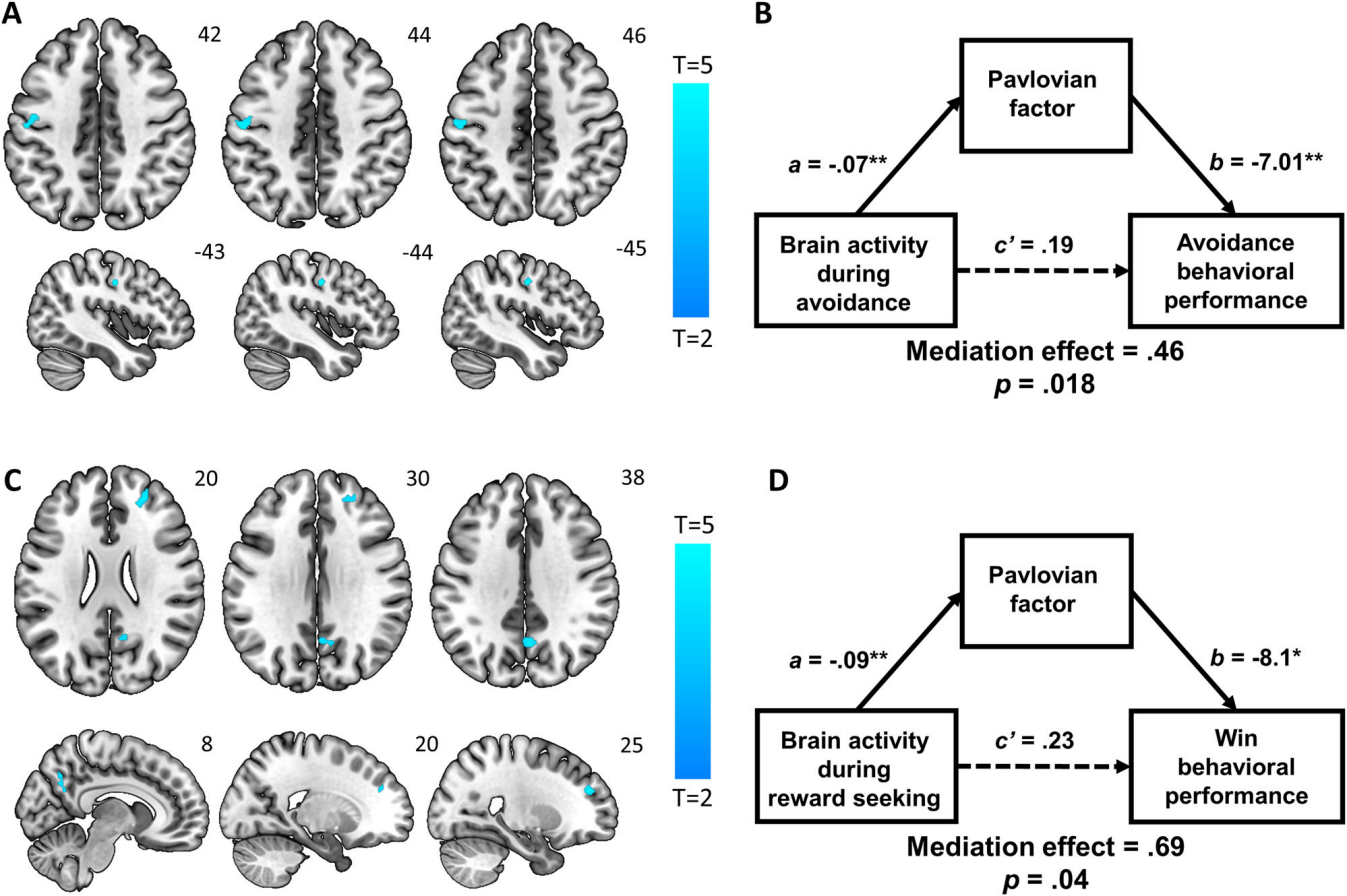
Neural correlates of pavlovian factor during pain avoidance and reward seeking. ***A***, Whole-brain multiple regression of avoid > 0 on the pavlovian factor negatively predicted activation in the left PrCG. ***B***, Mediation analysis: during pain avoidance, brain activation predicted performance accuracy, and this relationship was significantly and negatively mediated by the pavlovian factor. ***C***, During reward seeking (i.e., win > 0), the pavlovian factor negatively predicted activation in the right SFG and right PCC. ***D***, Mediation analysis: during monetary win trials, right SFG and right PCC activation predicted performance accuracy, and this relationship was significantly and negatively mediated by the pavlovian factor. *p < 0.05; **p < 0.01.

In whole-brain multiple regression with the pavlovian factor as the predictor showed a negative correlation with the activation of the left precentral gyrus (PrCG) during pain avoidance (Fig. 3*A*, Table 1).

Across participants, the pavlovian factor and performance accuracy of pain avoidance were negatively correlated (*r* = −0.35, *p* = 0.002). Furthermore, the regional activity correlates of individual variation in pavlovian factor were significantly correlated with the accuracy of pain avoidance performance (*r* = 0.24, *p* = 0.03). Thus, we performed a mediation analysis to examine the interrelationships between the activation in the PrCG, the pavlovian factor, and task performance during avoidance (Fig. 3*B*). The model in which the relationship between the brain activation and task performance was mediated by the pavlovian factor was significant. In other words, the greater the brain activity in the PrCG, the higher the task performance, and this relationship was significantly and negatively mediated by the pavlovian factor.

We next examined the neural correlates of the pavlovian factor during reward seeking, using the win > 0 contrast. The pavlovian factor predicted lower activation of the right superior frontal gyrus (SFG) and right PCC (Fig. 3*C*, Table 1). The reverse contrast did not yield any significant findings.

As task performance on win trials was correlated with both pavlovian factor (*r* = −0.34, *p* = 0.002) and SFG/PCC activation (*r* = 0.26, *p* = 0.01), we conducted a mediation analysis, which showed that the relationship between SFG/PCC activity and task performance was significantly and negatively mediated by the pavlovian factor (Fig. 3*D*).

#### Neural correlates of subjective impact of outcomes

**Figure 4.**
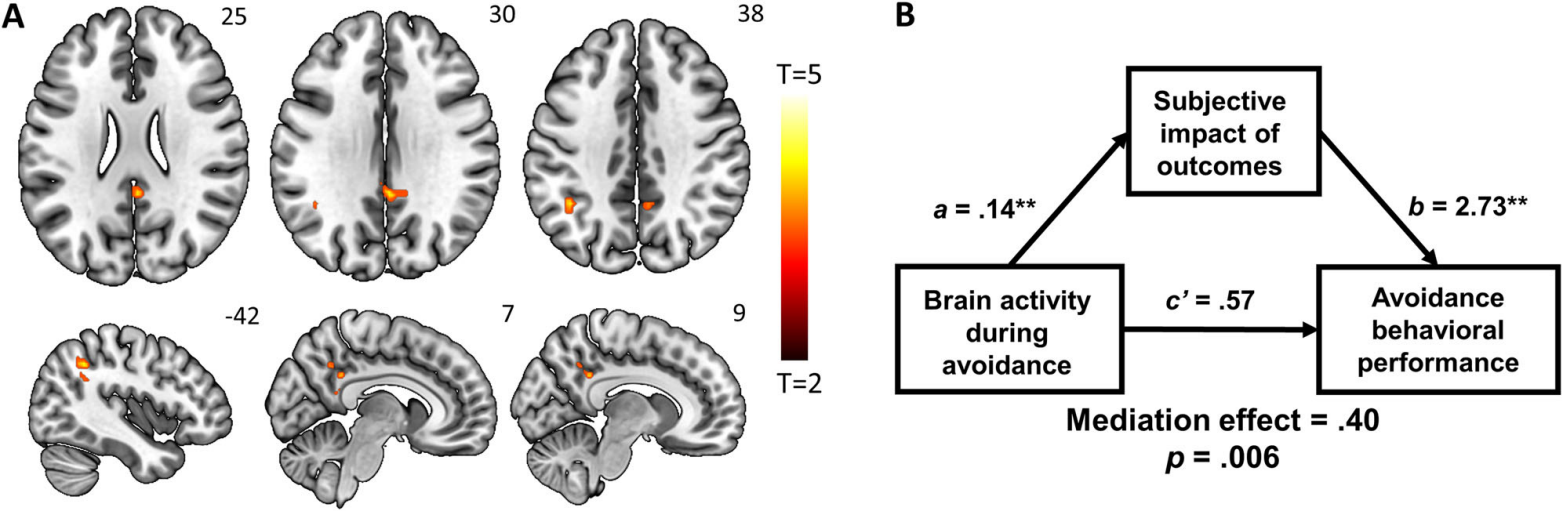
***A***, Whole-brain multiple regression of avoid > 0 on the subjective impact of outcomes positively predicted activations in the PCC and SPL. ***B***, Mediation analysis: during pain avoidance, brain activation positively predicted performance accuracy, and this relationship was significantly and positively mediated by the subjective impact of outcomes. *p < 0.05; **p < 0.01.

In a whole-brain multiple regression with the subjective impact of outcomes (*ρ*A) as the predictor of regional responses to avoid > 0, the PCC and left superior parietal lobule (SPL) showed activation in positive correlation with *ρ*A (Fig. 4*A*, Table 1).

Additionally, the performance accuracy during pain avoidance was positively correlated with both *ρ*A (*r* = 0.59, *p* < 0.001) and the parameter estimates of PCC/SPL activity (*r* = 0.48, *p* < 0.001). A mediation analysis showed that the higher the PCC/SPL activity during pain avoidance, the greater the performance accuracy, a relationship significantly and positively mediated by *ρ*A (Fig. 4*B*).

The same analyses applied to the *ρ*W during reward seeking (i.e., win > 0) did not yield any significant results.

#### Neural correlates of action bias

**Figure 5.**
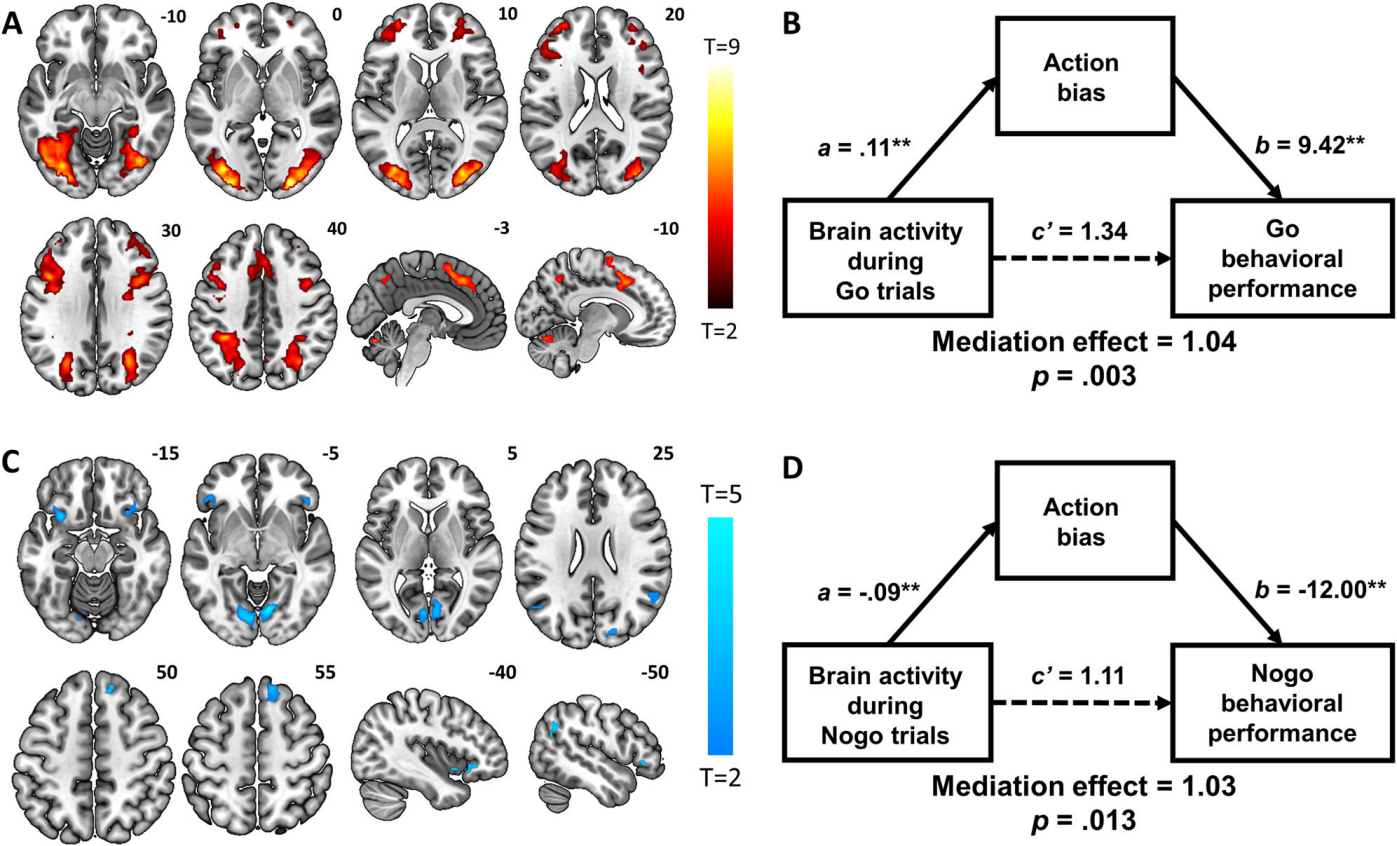
Neural correlates of action bias during go versus no-go trials (***A***) Whole-brain multiple regression of go > no-go on action bias positively predicted activations in the bilateral dlPFC; left superior frontal cortex; a cluster containing the SMA, pre-SMA, and dACC; and a large cluster containing the bilateral SPL, visual cortices, parahippocampal gyri, and cerebellum. ***B***, Mediation analysis: during go responses, brain activations positively predicted performance accuracy, and this relationship was significantly mediated by action bias. ***C***, Whole-brain multiple regression of go > no-go on action bias negatively predicted activations in the bilateral striate areas, bilateral superior temporal sulcus, bilateral AI, and right SFG. ***D***, Mediation analysis: during no-go responses, brain activations positively predicted performance accuracy, and this relationship was significantly mediated by action bias. *p < 0.05; **p < 0.01.

Finally, to determine the neural processes underlying the tendency to initiate an action, a whole-brain regression with the action bias as the predictor of activations to go > no-go contrast was conducted. Greater action bias was significantly correlated with higher activations in the bilateral dlPFC; left superior frontal cortex; a cluster containing the SMA, pre-SMA, and dACC; and a large cluster containing occipital cortices, SPL, parahippocampal gyri, and cerebellum (Fig. 5*A*, Table 2).

**Table 2.** Neural correlates of individual differences in RL parameters during action initiation

Region	MNI coordinates (mm)			Voxel *T*	Cluster *k*
	*X*	*y*	*z*		
Action bias during go > no-go					
Occipital cortex	25	−88	−2	9.62	4,843
	−30	−91	−4	9.14	
	−35	−86	6	8.56	
	40	−74	−10	7.85	
Cerebellum	−10	−74	−22	4.88	
SPL	−40	−44	46	8.05	
dlPFC	−30	54	16	7.78	1,069
	−40	12	33	7.39	
	−48	32	26	6.86	
	42	6	33	6.47	919
	30	−4	48	5.81	
SMA	0	22	46	5.93	446
	8	12	46	5.75	
	−8	12	50	5.70	
Dorsal ACC	−10	12	38	4.60	
Presupplementary motor area	−10	−1	68	4.15	
Action bias during no-go > go					
Striate area	10	−71	−4	7.47	359
	−8	−81	−2	6.62	
Superior temporal sulcus	−45	−56	33	5.67	58
	−48	−64	23	3.89	
	58	−54	33	4.54	53
	52	−54	26		
Insula	−32	14	−17	5.67	96
	−40	9	−10	5.10	
	−42	29	−7	4.95	
	48	32	−7	4.95	49
	30	16	−17		
SFG	12	36	56	5.19	59

We then extracted the parameter estimates for the regions identified in the multiple regression involving go > no-go and found a significant correlation with performance accuracy during go trials (*r* = 0.67, *p* < 0.001). The relationship between action bias and performance accuracy during go trials was also significant (*r* = 0.69, *p* < 0.001). A mediation analysis was thus performed to characterize the interrelationships between action bias, brain activation during go > no-go, and performance accuracy in go trials (Fig. 5*B*). We found that the model in which the relationship between brain activity in the regions identified and task performance during go trials was significantly and positively mediated by action bias.

Action bias also negatively predicted activations in the bilateral striate areas, bilateral superior temporal sulcus, bilateral anterior insula (AI), and right SFG (Fig. 5*C*, Table 2). We then extracted the parameter estimates for these regions and found a significant relationship with no-go behavioral performance (*r* = 0.51, *p* < 0.001). Finally, we conducted a mediation analysis, revealing that the relationship between brain activity during no-go responses and no-go behavioral performance was significantly and negatively mediated by action bias (Fig. 5*D*).

## Discussion

We investigated regional brain activations reflecting individual differences in four distinct RL components in learning to avoid pain and to seek reward: learning rate, subjective impact of outcomes, pavlovian factor, and action bias. The dACC, MCC, and left PoCG showed activity in a positive correlation with learning rate and predicted task performance of avoidance learning. The left PrCG showed activity in a negative correlation with the pavlovian factor during avoidance learning. Further, a cluster involving the right SFG and PCC showed activities in negative correlation with pavlovian factor during reward seeking. The subjective impact of outcomes was predictive of PCC and SPL activation and task performance during avoidance learning. Finally, we observed robust activations positively associated with action bias during go versus no-go trials in the dlPFC, SMA, pre-SMA, and dACC, SPL, and cerebellum. In contrast, there was a negative association between the right SFG activation and action bias. We discussed the main findings below.

### Behavioral performance

Participants showed highest performance accuracy in go-to-win (81.1%) and lowest (65.1%) in no-go-to-win trials, consistent with effects of reward on motivating approach behavior (Arias-Carrión and Pŏppel, 2007; Guitart-Masip et al., 2012; Hidi, 2016) and attenuating inhibitory control, especially when such reward is concurrently paired with a go response (Meyer et al., 2021). Next, we examined the learning rate that was significantly lower for win versus avoid trials. It is plausible that the relatively easy go-to-win condition represented a less-than-optimal opportunity for learning whereas the no-go-to-win condition may have been too challenging. Another possibility is that subjects may have been simply less inclined to change responses following errors during reward seeking relative to pain avoidance, resulting in a lower learning rate. More nuanced learning characteristics were revealed when we further separated learning based on the preceding outcomes. We found that participants showed better learning following favorable versus unfavorable outcomes, an asymmetry suggesting a bias toward learning from positive events (Frank et al., 2004; Cazé and van der Meer, 2013). Specifically, during learning to avoid shocks, participants exhibited superior learning after successfully avoiding pain compared with receiving shocks. During learning to gain money, participants similarly exhibited greater learning after successfully obtaining a monetary reward than achieving no reward. These findings are consistent with a previous PLGT study using monetary gain and loss (Oba et al., 2019). It is also worth noting that we randomly omitted half of the shocks to reduce head motion. Such omission may have affected learning due to weakened reinforcement as a result of the absence of pain. However, examining the feedback period, we found that the feedback with omitted shocks (i.e., display of the shock symbol without actual shocks) elicited similar regional activities implicated in pain response as compared with the feedback with actual shocks (data not shown). This suggested that the visual shock feedback alone was sufficient in activating the pain circuit and other brain regions subserving avoidance learning, and that the absence of shock likely exerted limited effects on learning.

As expected, we found a greater subjective impact of outcomes in reward versus avoid trials. This may have indicated participants’ greater valuation of monetary reward relative to shock avoidance. In the analyses of the stay/switch index, which assesses response certainty, we found that a higher subjective impact of outcomes was associated with greater response certainty in both reward and avoid trials. Thus, when subjects failed to learn the value of the stimuli, they appeared less certain in their responses, in line with previous reports (Myers et al., 2016; Robinson et al., 2021). The pavlovian factor, which quantifies the extent to which action values are influenced by stimulus values, independent of learning, was negatively associated with performance accuracy in both reward and avoid trials. Maximizing performance in the PLGT requires behavioral regulation. As stimulus value promotes go responses and reduces cognitive control (Dixon and Christoff, 2012), a heightened pavlovian factor can upset the regulatory processes in balancing between go and no-go.

### Neural correlates of individual differences in learning rates

Our findings showed that higher dACC activation was associated with a greater avoidance learning rate across subjects. This finding is broadly in line with previous reports of dACC activation during experimentally induced pain (Xu et al., 2020), fear conditioning (Büchel and Dolan, 2000), and aversive delay conditioning across multiple stimulus modalities (Sehlmeyer et al., 2009). The dACC also responds to errors in go/no-go (Orr and Hester, 2012), flanker (Gilbertson et al., 2021), continuous performance (Ichikawa et al., 2011) tasks, and during probabilistic learning (Holroyd et al., 2004), highlighting the region's involvement in learning. Error-related learning requires dopaminergic signaling (Allman et al., 2001; Holroyd and Yeung, 2012), at least partially supported by midbrain projections to dACC (Niv, 2007; Assadi et al., 2009; Holroyd and Yeung, 2012), and is known to exhibit individual differences in traits related to learning (Laakso et al., 2000; Reif and Lesch, 2003; Munafò et al., 2008). Further, in direct support of our work, μ-opioid receptor availability in the dACC was positively associated with individual differences in harm avoidance traits in healthy subjects (Tuominen et al., 2012). Despite such evidence, no work to our knowledge has explored how dACC may represent differences in avoidance learning characteristics across individuals. Thus, the current findings add to the literature by implicating the dACC in individual differences in associative learning involving avoidance of painful shocks.

Another region that showed activity in association with avoidance learning rate was the MCC. The MCC has been implicated in the anticipation and execution of avoidance response to pending noxious stimulation (Shackman et al., 2011). Motor neurons in the MCC may receive motivational information (e.g., pain, reward, error) to guide goal-directed behaviors (Vogt, 2016). In electroencephalographic recordings, the MCC appears to contribute to avoidance by evaluating the cost related to punishment in a Simon task (Cavanagh et al., 2014). During avoidance learning involving pain, the MCC was also found to respond to unexpected pain and pain avoidance (Jepma et al., 2022). Our findings of MCC activation in a positive association with avoidance learning rate contribute to this literature and substantiate the role of the MCC in tracking avoidance learning. It is worth pointing out that we observed higher dACC and MCC activities in association with individual variation in learning during pain avoidance but not reward seeking. Other factors potentially contributing to the intersubject variation in reward learning may not have been accounted for in our RL models. For instance, perseverance influences performance in difficult reward-learning tasks (Duckworth et al., 2007; Dale et al., 2018). With our participants’ average at 73% in performance accuracy (chance at 50%), perseverance as an additional factor may be needed to fully model reward learning.

### The neural correlates of individual differences in pavlovian factor

A higher pavlovian factor predicted SFG activation and worse performance in reward-seeking trials. The pavlovian factor refers to the extent to which actions are influenced by stimulus values, independent of learning. Thus, higher values of the pavlovian factor may have instigated go when no-go responses were required. The right SFG has been implicated in proactive control (Hu et al., 2016; see additional discussion of the role of the SFG below). The current findings thus extend the literature by showing the role of the right SFG in individual differences in balancing action initiation and inhibition, with hypoactivity negatively impacting behavioral performance during reward seeking.

Greater pavlovian factor was also correlated with lower left PrCG activation during pain avoidance, suggesting that stimulus value reduced PrCG activity. Mediation analysis further showed that the reduction in PrCG activity in turn decreased task performance, pointing to the possibility that the PrCG plays a role in avoidance behavior and that increased stimulus value may undermine this function of the region. Evidence for the involvement of the PrCG in the avoidance of aversive stimuli can be found in both human and animal research. For instance, stimulation of the polysensory zone of the PrCG elicited defensive movements in monkeys (Cooke and Graziano, 2004). An imaging study in humans showed left PrCG activity in a positive correlation with individual differences in avoidance response rates during the performance of an approach-avoidance task involving rewarding and aversive visual stimuli (Schlund et al., 2010). Other studies in healthy individuals showed higher left PrCG activity during response inhibition in a go/no-go task (Thoenissen et al., 2002) and during the avoidance of negative social scenes (Ascheid et al., 2019). As PrCG's activity may have been affected by stimulus value, it is plausible that the region receives inputs from brain structures associated with motivation and such inputs modulate PrCG activity. Our own previous work showed that the left PrCG exhibited functional connectivity with the medial orbitofrontal cortex, a region important for reward processing, and the pain-related periaqueductal gray. Importantly, these functional connectivities predicted positive alcohol expectancy in drinkers (Le et al., 2020). Thus, the PrCG's involvement in avoidance behavior may be affected by modulatory information from the reward and pain-related regions, which then help control motor responses accordingly. Taken together, left PrCG activity may support individual differences in the pavlovian factor via its interaction with the reward and punishment circuits.

### Neural correlates of individual differences in subjective impact of outcomes

The PCC showed activities reflecting individual differences in the subjective impact of outcomes during avoidance learning—a measure of stimulus-driven motivation to avoid painful shocks. There is abundant evidence for the involvement of the PCC in avoidance behavior. In rodents, PCC neuronal activities increased during behavioral avoidance during water maze navigation (Riekkinen et al., 1995) and during avoidance learning involving foot shocks (Vogt et al., 1991). Synaptic plasticity within the PCC was found to play a critical role in memory consolidation for avoidance (Pereira et al., 2002). PCC inactivation by muscimol following training impaired both memory and subsequent avoidance behavior (Souza et al., 2002). In humans, the PCC is likewise involved in defensive behavior during exposure to fear and threats (McNaughton and Corr, 2004). This proposal was supported by evidence of increased PCC activation to pain processing (Nielsen et al., 2005). Importantly, in our previous work, we showed PCC activation during response inhibition in positive correlation with individual differences in punishment sensitivity trait (Le et al., 2019), lending weight to the notion that the region subserves avoidance behavior.

The results of the mediation analysis demonstrated that PCC's involvement in avoidance behavior may have been mediated by learning of motivational values of outcomes. The better the learning by the individual, the greater the PCC activation, and the more consistent and higher behavioral performance. This finding suggested that the PCC may receive modulatory information from other brain regions that evaluate stimulus motivational values to influence behaviors. Indeed, tract-tracing studies in nonhuman primates found that the PCC is highly anatomically connected. The PCC has dense connections with other paralimbic and limbic structures including the hippocampal formation and parahippocampal cortex (Vogt and Pandya, 1987; Kobayashi and Amaral, 2007). The hippocampus may serve as the hub for the convergence between the set of limbic regions related to ventral stream processing (e.g., orbitofrontal, anterior cingulate, amygdala) and the PCC related to dorsal stream processing (Rolls, 2019). This convergence likely helps with the memory of “what” happens (i.e., outcomes) and “where” (i.e., context), both of which are crucial for avoidance learning.

### Neural correlates of individual differences in action bias

Finally, our whole-brain multiple regression with action bias as the predictor showed higher activity in the pre-SMA, SMA, dACC, and dlPFC. Both the pre-SMA and SMA have been implicated in action initiation, with reports of increased neuronal activities immediately before movement both in humans (Cunnington et al., 2005; Bortoletto and Cunnington, 2010) and nonhuman primates (Eccles, 1982; Nachev et al., 2008; Nakajima et al., 2009). The pre-SMA is a higher-order, cognitive motor region whose role likely includes the assessment of action value. Recent supporting evidence demonstrates that pre-SMA neurons in humans encoded option values during the decision phase of a two-armed bandit task (Aquino et al., 2023). Similarly, SMA neurons have been found to encode reward expectancy in monkeys performing eye movement tasks (Campos et al., 2005). Thus, both the pre-SMA and SMA offer a link between motivation and action. Here, individual pre-SMA/SMA activities were correlated both with action bias and go response accuracy to gain reward and avoid shocks, suggesting the regions may use motivational information (i.e., potential monetary gain or painful shocks) to execute appropriate motor activities.

Greater dACC activity was also associated with higher action bias. It is worth noting that this activity (*z* = 40) was located dorsal to the dACC cluster that represented individual avoidance learning rate (*z* = 20–40). With anatomical connectivity to the primary motor cortex and spinal cord (Matelli et al., 1991; Picard and Strick, 1996), the dACC is known to be involved in action initiation (Srinivasan et al., 2013). Other studies have suggested a more complex role, with dACC activity varying with motor and reward information during a pursuit/evasion task (Yoo et al., 2021) as well as with the probability of correct response selection during learning of stimulus–outcome association (Noonan et al., 2011).

We observed higher activation in bilateral dlPFC in association with the degree of action bias. The dlPFC is commonly implicated in executive control (Fuster, 2015) and, of specific relevance to the current findings, in representing and integrating goals and reward during decision-making (Miller and Cohen, 2001; Watanabe and Sakagami, 2007). As a hub where information about motivation, motor control, and desired outcomes converges, the dlPFC may have facilitated associative learning by relating visual cues to feedback (shock vs money) to determine required responses (go vs no-go). As with many other cortical regions, the dlPFC receives neuromodulatory inputs from the dopaminergic midbrain (Björklund and Dunnett, 2007), enabling RL via its influence on working memory, attention, and cognitive control processes (Ott and Nieder, 2019). In a study employing a simple rewarded reaction time task, dynamic causal modeling showed that reward information during goal-directed actions was the driving input to the dlPFC, which in turn modulated the activity of the ventral tegmental area and nucleus accumbens (Ballard et al., 2011). These findings support the dlPFC in reflecting individuals’ tendency to initiate goal-directed actions.

In contrast, the bilateral AI and right SFG exhibited activity in a negative association with action bias, suggesting their role in restraining actions. It is worth noting that this SFG cluster is dorsal (*z* = 55) to the region (*z* = 14) implicated in individual variation in pavlovian factor. A meta-analysis showed robust activation of the AI and SFG in response inhibition across multiple tasks (Zhang et al., 2017). Right SFG response to no-go inhibition was negatively correlated with trait impulsivity (Horn et al., 2003). Using a stop-signal task, another study associated greater right SFG activation with more efficient response inhibition and less motor urgency across subjects (Hu et al., 2016). These findings suggest right SFG's role in representing individual differences in cognitive control. Similarly, the AI activation during failed no-go responses in the go/no-go task was associated with individual variations in motor impulsivity and reactive aggression in healthy male adults (Dambacher et al., 2013). More broadly, AI volume showed a negative relationship with multiple impulsivity and compulsivity measures in individuals with alcohol use disorders (Grodin et al., 2017). Taken together, the AI and right SFG may represent an index of reduced action bias during goal-directed behaviors.

## Limitations

The current study has several potential limitations. First, we used electric shocks and monetary gains to motivate learning associated with pain avoidance and reward seeking. Across subjects, we observed a greater learning rate for pain avoidance relative to reward seeking. It is plausible that this difference reflected higher saliency for electric shocks versus money. To explore this possibility, we analyzed the skin conductance data during feedback of money versus shocks (including both actual and omitted shocks). The skin conductance responses did not differ significantly across the two conditions (data not shown), suggesting comparable saliency between the two contingencies. Despite this finding, we cannot rule out the possibility that learning was biased by the use of the two distinct motivational outcomes. Second, to reduce the effects of head motion, we only delivered the shocks half of the time the shock feedback was displayed. Learning may have been affected by the absence of shock delivery. However, we found that feedback with omitted and actual shocks both elicited robust activations in regions previously reported to be responsive to experimentally induced pain (Xu et al., 2020). Thus, negative feedback with omitted shocks was likely sufficiently salient to evoke activations indicative of pain or pain anticipation.

## Conclusions

The current work investigated the neural correlates of individual differences in RL. Learning was facilitated by positive outcomes and hindered by the excessive influence of stimulus value. Our imaging results shed light on the neural correlates of various RL metrics, showing distinct roles of the medial frontal cortex, including the ACC, in representing avoidance learning rate, pavlovian factor, and action bias. We confirmed the involvement of the PCC and PrCG in avoidance learning by demonstrating their activities in correlation with individual differences in learning characteristics. These findings may have important implications for the understanding of mental disorders that manifest as dysfunctional RL.

## Synthesis

Reviewing Editor: Niko Busch, Westfalische Wilhelms-Universitat Munster Decisions are customarily a result of the Reviewing Editor and the peer reviewers coming together and discussing their recommendations until a consensus is reached. When revisions are invited, a fact-based synthesis statement explaining their decision and outlining what is needed to prepare a revision will be listed below. The following reviewer(s) agreed to reveal their identity: Michel-Pierre Coll.

# Synthesis

Your manuscript has been reviewed by two experts on reinforcement learning. The reviewers' comments have been included at the bottom of this letter. Both reviews were largely positive, highlighting the merits of this study. However, the reviewers have a few requests for further clarification, particularly regarding the methods and results, and for additional control analyses. I encourage you to address each of the reviewers' points carefully. Finally, I would like to ask you to consider making data and code publicly available.

# Reviewer 1

Thank you for the opportunity to review this interesting paper. The authors used fMRI during a reward/punishment GoNoGo task with a learning component. They attempted to understand individual differences in the neural correlates of separable RL components, using a task that neatly orthogonalizes approach/avoid and reward/punishment contingencies. While the general topic of neural correlates of RL components has been well-explored, the characterization of individual differences in a relatively large (82 adults) sample, and in this specific context, is novel. The methodology appears sound. A relatively high spatiotemporal resolution fMRI sequence was used and sources of noise not often considered in task fMRI analyses (respiration and heart rate) were regressed out. The paper is very well-written and clear. These results would be of broad interest to the eNeuro readership. I believe this paper is worthy of publication in eNeuro; my comments and suggestions are below.

Abstract: "subjective impact of outcomes" is a little vague - can you please clarify/operationalize? I see it is done in the manuscript but it would benefit from being added to the abstract.

The age range appears to be quite large (SD = 11.2 years) - can you please include the full age range of participants? Additionally, the regression analyses control for age - can you describe the effects of age on these primary outcomes in a supplemental analysis?

Likewise, were there significant sex differences in the RL metrics/associated brain activities?

Was there any self-reported data on attitudes towards/experience with pain?

Are there any data on participants baseline ratings of how positive they find the $1 reward relative to how negative they find the shock? How do we know that any learning biases between reward and punishment are due to fundamental differences if reward versus punishment learning, rather than one condition being much more salient than another in this specific task?

The fMRI cluster significance thresholding is appropriate - could the authors please report the minimum cluster size they selected?

Can you add a sentence or two in the discussion on how the 50% shock delivery during shock feedback may have affected the behavioral outcomes here?

Line 617 should be 'tract-tracing'

# Reviewer 2

This manuscript reports the results of an fMRI study in which 82 participants performed an approach/avoidance learning task. In this task, participants had to either press or avoid pressing a button to obtain a probabilistically related reward (Go win, No-go win) or to avoid a probabilistically related painful shock (Go avoid, No-go avoid). Different Q-learning models were fitted on participants' choice behaviour, and the final best-fitting model included eight free parameters, that is, four different learning rates (2 money/shock x 2 positive/negative prediction errors), 2 subjective outcome modifiers, a Pavlovian factor and an action bias parameter. fMRI data were modelled at the first level using a GLM, and three contrasts were performed: Avoid vs 0, Win vs 0, Go vs No go. Using these contrasts, several group-level regressions were performed between the different parameters of the learning models and the parametric maps of the contrasts.

This is an interesting study with a relatively large sample size and a well-thought-out learning paradigm; the attempt to link learning parameters with brain activity could provide valuable information on the neural bases of individual differences in learning. However, the specific details of the various analyses are hard to understand from the text, and if I understood correctly, I have several concerns regarding the analytical approach.

Major points

1. Clear reporting of the analyses. The results presented are challenging to evaluate due to the complexity and intricacy of the analyses performed. To facilitate a clearer understanding and enable a thorough assessment, I recommend that the authors provide a more detailed and precise description of their analytical methods. This could be achieved either within the main text or as part of supplementary materials. For instance, the following are specific examples of analyses that would benefit from a more explicit and detailed explanation:

1.1 The winning model employs four different learning rates. Yet, on page 19, only two whole-brain regressions are reported with avoidance and reward learning rates. It is unclear if the positive/negative PE aspect of the rates was considered or not here and, if not, the reason for doing so.

1.2. Were only the onset of the cues included in the GLM? If so, were the Go/No Go regressors used in a second model? It is also unclear why temporal derivatives were included in the model. Please indicate all the regressors entered in the GLM and the rationale for the contrast at the first level. If only the Avoid/Reward regressors were included in a model, it seems hard to interpret the Avoid vs 0 and Win contrast vs 0 since these contrasts are against the unmodelled baseline, but in this case, this baseline will include many things (feedback, button presses, shocks...), so the nature of the activity derived from this contrast is hard to interpret. Taking into account correct answers and mistakes in the GLM could also be worthwhile since different activities are probably associated with successful or failed trials.

1.3. The reference provided for the mediation analysis leads to a frustrating thread of other references (see Le et al., 2019, which points to Ide & Li, 2014, which points to Ide & Li, 2011) with similar text but not enough details to understand the analysis. Please try to make the paper as self-contained as possible or point to a single complete explanation. It is also unclear which brain activity was included in the mediation analyses. The text mentions that the parameters from the whole brain regression were extracted but does not mention how the multiple parameters were entered into a single mediation analysis. Were the parameters averaged? If so, across all regions, only significant regions, ROIs?

1.4. Response time is mentioned in the analysis but not reported in the results. Differences in response times are likely in this paradigm and could have an important impact on the assessment of BOLD responses (see Mumford et al., 2023) and the relationship between performance and BOLD.

Mumford, J. A., Bissett, P. G., Jones, H. M., Shim, S., Rios, J. A. H., & Poldrack, R. A. (2023). The response time paradox in functional magnetic resonance imaging analyses. Nature Human Behaviour, 1-12.

3. Description of the variables used in regression models. It is difficult to assess the validity of the between-participants regression analyses without knowing the underlying distribution of the individual differences in the sample. Were there actually evident individual differences between the participants? If parameters were bounded, ceiling effects could be present, and if not, outliers might contribute to the relationships. Please display or report the distribution of all learning parameters used as independent variables in the regression models and the correlation matrix of the different parameters and behavioural variables. Similarly, fMRI data is only reported in terms of its relationship with learning parameters. However, it seems necessary to report the results of the univariate tests for the different contrasts to confirm that the BOLD data conforms to what would be expected for Avoidance vs reward and go vs no activity.

4. Covariates and confounds. Sex and age are entered in the models as covariates, but other, perhaps more important covariates are not considered, including response time, and movement (i.e. framewise displacement), number of shocks received, which could confound the relationship between brain activity and the learning parameters. The relationship between these variables and the different parameters should be reported, and if non-negligible, they could be considered covariates.

5. Reliability of the variable entered in the regression models. One of the central issues with the analytical approach is that both BOLD activity (e.g. Plichta et al., 2012) and learning parameter estimates (Vrizzi et al., 2023) are measured with low reliability. It is, therefore, unclear how strong of a relationship between these variables could be observed across individuals, which raises the issue of the power of the analysis despite the relatively large sample size (Marek et al., 2022). Perhaps a more convincing demonstration of the relationship between the learning parameters and brain activity would be to show an out-of-sample generalization of the model weights, for example, by using cross-validation. This would demonstrate that the regions identified can actually predict individual differences in the learning parameters.

Marek, S., Tervo-Clemmens, B., Calabro, F. J., Montez, D. F., Kay, B. P., Hatoum, A. S., ... & Dosenbach, N. U. (2022). Reproducible brain-wide association studies require thousands of individuals. Nature, 603(7902), 654-660.

Plichta, M. M., Schwarz, A. J., Grimm, O., Morgen, K., Mier, D., Haddad, L., ... & Meyer-Lindenberg, A. (2012). Test-retest reliability of evoked BOLD signals from a cognitive-emotive fMRI test battery. Neuroimage, 60(3), 1746-1758.

Vrizzi, S., Najar, A., Lemogne, C., Palminteri, S., & Lebreton, M. (2023). Comparing the test-retest reliability of behavioral, computational and self-reported individual measures of reward and punishment sensitivity in relation to mental health symptoms.

Minor points

- The decision to deliver a shock to only half of the negatively reinforced trials could be better justified, and its impact on learning should be considered. Were the prediction errors considered equivalent for negative feedback compared to negative feedback + shock?

- I wonder if "performance" is the best term to describe expected responses since these are directly contingent on learning, and depending on the reinforcement schedule, one could provide more "inaccurate" responses just because they did not have the opportunity they were exposed to the low probability outcome.

- Bar graphs would be more informative if they showed the distribution of the observations with an overlaid dot plot.

- Since different outcome types were used, the fact that positive rewards led to higher learning rates might be due to different subjective values of the outcomes (people probably care more about gaining money than avoiding mild shocks). I think the interpretation of this result should be nuanced to take this into account.

Typos, formatting, etc.

- The second sentence of the abstract introduces several technical terms that are not defined and could be made more accessible.

- Shocks are incorrectly referred to as losses in some places (e.g. p.10).

- Since the regression models were not assessed out of sample, the term "prediction" to describe a significant in-sample association should be avoided.

-On page 4. Not clear what regional responses are and how they differ from neuronal responses.

- Figure 2 and upward, the * are not defined.

## Author Response

Response to Reviewers # Synthesis Your manuscript has been reviewed by two experts on reinforcement learning. The reviewers' comments have been included at the bottom of this letter. Both reviews were largely positive, highlighting the merits of this study. However, the reviewers have a few requests for further clarification, particularly regarding the methods and results, and for additional control analyses. I encourage you to address each of the reviewers' points carefully. Finally, I would like to ask you to consider making data and code publicly available.

Response: We thank the editor the opportunity to revise our manuscript. Our data have uploaded to NIMH Data Archive and are available according to the NIH guidelines. The code is freely available as per the journal's instructions. We thank the reviewers for the constructive comments and have revised the manuscript accordingly. All changes are highlighted in yellow in the manuscript. We have addressed each comment in detail below. # Reviewer 1 Thank you for the opportunity to review this interesting paper. The authors used fMRI during a reward/punishment GoNoGo task with a learning component. They attempted to understand individual differences in the neural correlates of separable RL components, using a task that neatly orthogonalizes approach/avoid and reward/punishment contingencies. While the general topic of neural correlates of RL components has been well-explored, the characterization of individual differences in a relatively large (82 adults) sample, and in this specific context, is novel. The methodology appears sound. A relatively high spatiotemporal resolution fMRI sequence was used and sources of noise not often considered in task fMRI analyses (respiration and heart rate) were regressed out. The paper is very well-written and clear. These results would be of broad interest to the eNeuro readership. I believe this paper is worthy of publication in eNeuro; my comments and suggestions are below.

Abstract: "subjective impact of outcomes" is a little vague - can you please clarify/operationalize? I see it is done in the manuscript but it would benefit from being added to the abstract.

Response: We apologize for not including the definition of the term in the abstract. A brief explanation has now been added (pg. 1).

The age range appears to be quite large (SD = 11.2 years) - can you please include the full age range of participants? Additionally, the regression analyses control for age - can you describe the effects of age on these primary outcomes in a supplemental analysis? Response: The age range was 21-59 years. As the reviewer suggested, we examined the relationship between age and the learning parameters. Age showed no significant relationship with avoidance learning rate (r = -.17, p = .012), reward learning rate (r = -.18, p = .10), Pavlovian factor (r = .21, p = .06), or action bias (r = -.03, p = .78). However, age was significantly correlated with subjective impact of outcomes during avoidance (r = -.34, p = .002) and subjective impact of outcomes during reward learning (r = -.34, p = .002). As the journal does not allow supplemental material, we were unable to report these results as such.

Likewise, were there significant sex differences in the RL metrics/associated brain activities? Response: Sex differences were not significant for avoidance learning rate (p = .24), reward learning rate (p = .24), Pavlovian factor (p = .09), action bias (p = .66), or subjective impact of outcomes during reward seeking (p = .60). However, men showed significantly greater subjective impact of outcomes during avoidance than women (p = .01). Whole-brain two-sample t-tests did not reveal any significant differences in brain activations between women and men.

Was there any self-reported data on attitudes towards/experience with pain? Response: All participants reported their pain sensitivity via the Pain Sensitivity Questionnaire. The sensitivity ratings did not show a significant relationship with avoidance learning rate (p = .54), Pavlovian factor (p = .46), subjective impact of outcomes (p's > .20), or action bias (p = .11).

Are there any data on participants baseline ratings of how positive they find the $1 reward relative to how negative they find the shock? How do we know that any learning biases between reward and punishment are due to fundamental differences if reward versus punishment learning, rather than one condition being much more salient than another in this specific task? Response: We appreciate that reviewer raised this issue. In our pilot studies, we did attempt to evaluate how participants perceived the saliency or intensity of $1 reward vs. electric shocks. The participants were unable to clearly judge how the two outcomes compared. Nonetheless, to address the reviewer's point, we analyzed the skin conductance data to examine potential differences in responses to the feedback of money vs. shocks (including both actual and omitted shocks). We found that the skin conductance responses did not differ significantly across the two conditions (p = .19), suggesting comparable saliency between the two contingencies. We have also discussed this as a potential limitation of the study (pg. 30) The fMRI cluster significance thresholding is appropriate - could the authors please report the minimum cluster size they selected? Response: Our minimum cluster size was 30 voxels. We have provided this info in the revision (pg. 12).

Can you add a sentence or two in the discussion on how the 50% shock delivery during shock feedback may have affected the behavioral outcomes here? Response: As suggested, we have added the following to the Discussion (pg. 22): "It is also worth noting that we randomly omitted half of the shocks to reduce head motion. Such omission may have affected learning due to weakened reinforcement as a result of the absence of pain. However, examining the feedback period, we found that the feedback with omitted shocks (i.e., display of the shock symbol without actual shocks) elicited similar regional activities implicated in pain response as compared to the feedback with actual shocks (data not shown). This suggested that the visual shock feedback alone was sufficient in activating the pain circuit and other brain regions subserving avoidance learning and that the absence of shock likely exerted limited effects on learning." Line 617 should be 'tract-tracing' Response: We apologize for the typo. It has now been corrected. # Reviewer 2 This manuscript reports the results of an fMRI study in which 82 participants performed an approach/avoidance learning task. In this task, participants had to either press or avoid pressing a button to obtain a probabilistically related reward (Go win, No-go win) or to avoid a probabilistically related painful shock (Go avoid, No-go avoid). Different Q-learning models were fitted on participants' choice behaviour, and the final best-fitting model included eight free parameters, that is, four different learning rates (2 money/shock x 2 positive/negative prediction errors), 2 subjective outcome modifiers, a Pavlovian factor and an action bias parameter. fMRI data were modelled at the first level using a GLM, and three contrasts were performed: Avoid vs 0, Win vs 0, Go vs No go. Using these contrasts, several group-level regressions were performed between the different parameters of the learning models and the parametric maps of the contrasts.

This is an interesting study with a relatively large sample size and a well-thought-out learning paradigm; the attempt to link learning parameters with brain activity could provide valuable information on the neural bases of individual differences in learning. However, the specific details of the various analyses are hard to understand from the text, and if I understood correctly, I have several concerns regarding the analytical approach.

Major points 1. Clear reporting of the analyses. The results presented are challenging to evaluate due to the complexity and intricacy of the analyses performed. To facilitate a clearer understanding and enable a thorough assessment, I recommend that the authors provide a more detailed and precise description of their analytical methods. This could be achieved either within the main text or as part of supplementary materials. For instance, the following are specific examples of analyses that would benefit from a more explicit and detailed explanation:

1.1 The winning model employs four different learning rates. Yet, on page 19, only two whole-brain regressions are reported with avoidance and reward learning rates. It is unclear if the positive/negative PE aspect of the rates was considered or not here and, if not, the reason for doing so.

Response: We apologize for the lack of details in the Methods section and have provided additional clarification per the reviewer's comments. Regarding the learning rates, as the reviewer pointed out, we did indeed have four types, including one for each of win trials with positive outcomes, win trials with negative outcomes, avoid trials with positive outcomes, and avoid trials with negative outcomes. To perform the whole-brain multiple regression for win trials and avoid trials, we averaged the two learning rates (i.e., learning from positive outcomes and learning from negative outcomes) for each of the two trial types. Consequently, we ended up with whole-brain multiple regressions with the two averaged learning rates, one for win trials and one for avoid trials, as the predictors. Please note that the learning rate in each of the four categories was positive in all participants regardless whether the outcomes were negative or positive, thus allowing us to calculate the means. This information has now been incorporated in the Methods section (pg. 12).

1.2. Were only the onset of the cues included in the GLM? If so, were the Go/No Go regressors used in a second model? It is also unclear why temporal derivatives were included in the model. Please indicate all the regressors entered in the GLM and the rationale for the contrast at the first level. If only the Avoid/Reward regressors were included in a model, it seems hard to interpret the Avoid vs 0 and Win contrast vs 0 since these contrasts are against the unmodelled baseline, but in this case, this baseline will include many things (feedback, button presses, shocks...), so the nature of the activity derived from this contrast is hard to interpret. Taking into account correct answers and mistakes in the GLM could also be worthwhile since different activities are probably associated with successful or failed trials.

Response: In the GLM, we modeled activations associated with the cue and feedback periods separately. The cue regressors were onsets of win and avoid trials. For the feedback period, we included the onsets of monetary win, shock, and null feedback as the regressors. In the current work, we only reported findings associated with the cue period. Thus, the results of the cue period were not confounded by brain activities related to win or shock feedback.

As the reviewer suggested, we examined the GLMs with correct and incorrect trials separated. The multiple regressions with correct trials yielded similar results to the findings we reported in the manuscript. No materially significant deviations were observed. Next, we performed multiple regressions using Avoid correct trials > Avoid incorrect trials and Win correct trials > Win incorrect trials contrasts. The multiple regression of Win correct trials > Win incorrect trials contrast with Pavlovian factor as the predictor showed that greater Pavlovian factor was associated with higher activation in the right middle frontal gyrus and right insula (Fig. R1A).

The multiple regression of Avoid correct trials > Avoid incorrect trials contrast with the subjective impact of outcomes during avoidance as the predictor showed higher activation in the right inferior parietal lobule (Fig. R1B). The multiple regressions with the two learning rates or the subjective impact of outcomes during reward learning as the predictors did not show any significant results in either direction.

Figure R1: Multiple regressions with Pavlovian factor (A) and subjective impact of outcomes (B) as the predictor of brain activation from the contrast Win correct trials > Win incorrect trials and Avoid correct trials > Avoid incorrect trials, respectively.

1.3. The reference provided for the mediation analysis leads to a frustrating thread of other references (see Le et al., 2019, which points to Ide & Li, 2014, which points to Ide & Li, 2011) with similar text but not enough details to understand the analysis. Please try to make the paper as self-contained as possible or point to a single complete explanation. It is also unclear which brain activity was included in the mediation analyses. The text mentions that the parameters from the whole brain regression were extracted but does not mention how the multiple parameters were entered into a single mediation analysis. Were the parameters averaged? If so, across all regions, only significant regions, ROIs? Response: We apologize for the unclear citations. Our mediation analysis follows the method of MacKinnon et al., 2007. Thus, we have now cited this work as our main reference.

Regarding the brain activity in each mediation analysis, we used the parameter estimates extracted from all the regions identified in the relevant multiple regression. For instance, in the mediation analysis depicted in Fig. 2B, we used the brain activity from all the significant regions identified from the multiple regression with the learning rate as the predictor (i.e., the dorsal anterior cingulate cortex, mid-cingulate cortex, left postcentral gyrus, and left superior temporal sulcus). The brain activity represented the averaged activation across all voxels in these regions. We have provided additional details in the Methods section for more clarity (pg. 13).

1.4. Response time is mentioned in the analysis but not reported in the results. Differences in response times are likely in this paradigm and could have an important impact on the assessment of BOLD responses (see Mumford et al., 2023) and the relationship between performance and BOLD.

Mumford, J. A., Bissett, P. G., Jones, H. M., Shim, S., Rios, J. A. H., & Poldrack, R. A. (2023). The response time paradox in functional magnetic resonance imaging analyses. Nature Human Behaviour, 1-12.

Response: Per the reviewer's suggestion, we examined the relationship between response time (RT) and the learning parameters as well as their neural correlates. Action bias was negatively correlated with both Go-to-win (r = -.32, p = .004) and Go-to-avoid (r = -.27, p = .016) RT. No significant relationships were observed between RT and learning rates or subjective impact of outcomes (p's > .06). Pavlovian factor showed a significant relationship with RT of reward trials (r = -.26, p = .018). Next, we sought to confirm that RT did not significantly drive our imaging results. To accomplish this, we constructed a new GLM with the same regressors and with RT included as a parametric modulator for go trials. First, we built the contrast Avoid RT > 0 and Win RT > 0 then conducted multiple regressions with the learning parameters as the predictors. Avoidance learning rate showed no significant positive relationship with activation from the Avoid RT > 0 contrast. There was a negative relationship with activation in the medial superior frontal gyrus. The reward learning rate did not exhibit a significant relationship with activation from the Win RT > 0 contrast in either direction. The Pavlovian factor was negatively associated with activation in the primary visual cortex from the Avoid RT > 0 contrast. There was no significant relationship with the multiple regressions involving the subjective impact of outcomes. As the results from the RT multiple regressions did not overlap with those from our original findings, RT did not play a significant role in our identification of the neural correlates of reinforcement learning.

3. Description of the variables used in regression models. It is difficult to assess the validity of the between-participants regression analyses without knowing the underlying distribution of the individual differences in the sample. Were there actually evident individual differences between the participants? If parameters were bounded, ceiling effects could be present, and if not, outliers might contribute to the relationships. Please display or report the distribution of all learning parameters used as independent variables in the regression models and the correlation matrix of the different parameters and behavioural variables. Similarly, fMRI data is only reported in terms of its relationship with learning parameters. However, it seems necessary to report the results of the univariate tests for the different contrasts to confirm that the BOLD data conforms to what would be expected for Avoidance vs reward and go vs no activity.

Response: Per the reviewer's comment, we have provided the histograms and correlation matrix of learning parameters below.

Figure R2: Histograms of the six learning parameters: reward learning rate, avoidance learning rate, Pavlovian factor, subjective impact of outcomes during reward learning, subjective impact of outcomes during avoidance learning, and action bias.

It is worth noting that we identified two subjects with high Pavlovian factor. We thus conducted the analyses involving the Pavlovian factor without the two subjects. The results did not materially change from our reported findings. As a result, we opted to keep the original findings with the inclusion of the two subjects.

Further, we conducted the univariate tests as the reviewer recommended. The contrast Win > Avoid showed significant activation in multiple regions including the medial orbitofrontal cortex, putamen, superior frontal gyrus, insula, superior temporal sulcus, postcentral gyrus, and cerebellum (Fig. R3A). Notably, the medial orbitofrontal cortex and putamen have been implicated in reward processing and reward learning (Haber and Knutson, 2010; Hori et al., 2009; Rolls, 2004). The contrast Avoid > Win showed significant activation in the bilateral anterior insula and pre-SMA (Fig. R3B), both of which have been found to play a role in avoidance behavior (Lynn et al., 2016; Palminteri et al., 2012; Samanez-Larkin et al., 2008). These results, therefore, are consistent with our current understanding of the brain circuits involved in reinforcement learning.

Figure R3: Univariate tests showed significant activations for contrast (A) Win > Avoid and (B) Avoid > Win.

4. Covariates and confounds. Sex and age are entered in the models as covariates, but other, perhaps more important covariates are not considered, including response time, and movement (i.e. framewise displacement), number of shocks received, which could confound the relationship between brain activity and the learning parameters. The relationship between these variables and the different parameters should be reported, and if non-negligible, they could be considered covariates.

Response: Per the reviewer's comment, we examined the relationship of the learning parameters with framewise displacement (FD) and number of total shocks. No significant correlation was observed with FD (p's > .11). The number of total shocks also did not show a significant relationship with avoidance learning rate (p = .15) but was significantly correlated with Pavlovian factor (r = .30, p = .007), subjective impact of outcomes during avoidance (r = -.48, p < .001), and action bias (r = .22, p = .04). Thus, for the multiple regressions involving avoidance learning, we included the total number of shocks received as a covariate. The multiple regression with avoidance learning rate as the predictor of activities from the Avoid > 0 contrast yielded positive and significant activation in the left postcentral gyrus, anterior cingulate cortex, left superior temporal sulcus, and midcingulate cortex. These results did not materially deviate from our reported findings. Similarly, Pavlovian factor showed a significant and negative relationship with activation in the left precentral gyrus during avoidance learning as well as a negative relationship with activation in the superior frontal gyrus and right posterior cingulate cortex during reward learning. The subjective impact of outcomes showed a significant and positive relationship with activation in the posterior cingulate cortex and left superior parietal lobule during avoidance learning. These results have now been reported in the manuscript as a direct replacement of previous reported findings. The Methods section has also been modified accordingly (pg. 12).

5. Reliability of the variable entered in the regression models. One of the central issues with the analytical approach is that both BOLD activity (e.g. Plichta et al., 2012) and learning parameter estimates (Vrizzi et al., 2023) are measured with low reliability. It is, therefore, unclear how strong of a relationship between these variables could be observed across individuals, which raises the issue of the power of the analysis despite the relatively large sample size (Marek et al., 2022). Perhaps a more convincing demonstration of the relationship between the learning parameters and brain activity would be to show an out-of-sample generalization of the model weights, for example, by using cross-validation. This would demonstrate that the regions identified can actually predict individual differences in the learning parameters.

Marek, S., Tervo-Clemmens, B., Calabro, F. J., Montez, D. F., Kay, B. P., Hatoum, A. S., ... & Dosenbach, N. U. (2022). Reproducible brain-wide association studies require thousands of individuals. Nature, 603(7902), 654-660.

Plichta, M. M., Schwarz, A. J., Grimm, O., Morgen, K., Mier, D., Haddad, L., ... & Meyer-Lindenberg, A. (2012). Test-retest reliability of evoked BOLD signals from a cognitive-emotive fMRI test battery. Neuroimage, 60(3), 1746-1758.

Vrizzi, S., Najar, A., Lemogne, C., Palminteri, S., & Lebreton, M. (2023). Comparing the test-retest reliability of behavioral, computational and self-reported individual measures of reward and punishment sensitivity in relation to mental health symptoms.

Response: We do not feel that we have a large enough dataset to perform cross-validation, for instance, by splitting the sample in halves. However, we wish to emphasize that our results were obtained with a corrected threshold, following current reporting standards. We have described this issue as a limitation in the revision (pg. 31).

Minor points - The decision to deliver a shock to only half of the negatively reinforced trials could be better justified, and its impact on learning should be considered. Were the prediction errors considered equivalent for negative feedback compared to negative feedback + shock? Response: In our models, prediction errors were considered equivalent for omitted shock feedback vs. actual shock feedback. To explore the activations to feedback associated with omitted shocks and with actual shocks, we examined the contrasts Actual shock feedback > Null feedback (Fig. R4A) and Omitted shock feedback > Null feedback (Fig. R4B). Both contrasts produced robust activations in regions reported in past imaging meta-analyses to be responsive to experimentally induced pain (Duerden and Albanese, 2013; Xu et al., 2020). Thus, this finding suggests that negative feedback with omitted shocks was sufficiently salient to evoke activations indicative of pain or anticipation of pain. Furthermore, the shock omission was randomized to ensure participants could not predict shock delivery. Given the potentially significant artifacts induced by head motion with excessive number of shocks, we believe the shock omission was an appropriate feature of the design. We have now discussed this point in the revision (pg. 22, 30).

Figure R4: Activations associated with feedback with (A) actual shocks and (B) omitted shocks.

- I wonder if "performance" is the best term to describe expected responses since these are directly contingent on learning, and depending on the reinforcement schedule, one could provide more "inaccurate" responses just because they did not have the opportunity they were exposed to the low probability outcome.

Response: We appreciate reviewer's concern. Unfortunately, we could not think of a better term to describe these behavioral outcomes and would be happy to adopt any suggestions reviewer may have.

- Bar graphs would be more informative if they showed the distribution of the observations with an overlaid dot plot.

Response: Per the reviewer's suggestion, we have modified the graphs (pg. 7).

- Since different outcome types were used, the fact that positive rewards led to higher learning rates might be due to different subjective values of the outcomes (people probably care more about gaining money than avoiding mild shocks). I think the interpretation of this result should be nuanced to take this into account.

Response: We found that learning rate was higher for avoid relative to win trials and an opposite pattern for subjective impact of outcomes. We have now included this interpretation in our Discussion section (pg. 23).

Typos, formatting, etc.

- The second sentence of the abstract introduces several technical terms that are not defined and could be made more accessible.

Response: We have now clarified the term subjective impact of outcomes and Pavlovian factor (pg. 1).

- Shocks are incorrectly referred to as losses in some places (e.g. p.10).

Response: We apologize for the typos which have now been corrected.

- Since the regression models were not assessed out of sample, the term "prediction" to describe a significant in-sample association should be avoided.

Response: We have now replaced the term "prediction" with "association".

-On page 4. Not clear what regional responses are and how they differ from neuronal responses.

Response: We have modified our wording to clarify "neuronal responses" as neurons' activity recorded at the neuronal level and "regional activation" as brain activity acquired via functional imaging.

- Figure 2 and upward, the * are not defined.

Response: We apologize for the omission. The definition has now been added to the figure legends.

- References

Duerden, E.G., Albanese, M.-C., 2013. Localization of pain-related brain activation: A meta-analysis of neuroimaging data. Hum. Brain Mapp. 34, 109-149. https://doi.org/10.1002/hbm.21416

Haber, S.N., Knutson, B., 2010. The reward circuit: Linking primate anatomy and human imaging. Neuropsychopharmacology 35, 4-26. https://doi.org/10.1038/npp.2009.129

Hori, Y., Minamimoto, T., Kimura, M., 2009. Neuronal Encoding of Reward Value and Direction of Actions in the Primate Putamen. J. Neurophysiol. 102, 3530-3543. https://doi.org/10.1152/jn.00104.2009

Lynn, M.T., Demanet, J., Krebs, R.M., Van Dessel, P., Brass, M., 2016. Voluntary inhibition of pain avoidance behavior: an fMRI study. Brain Struct. Funct. 221, 1309-1320. https://doi.org/10.1007/s00429-014-0972-9

Palminteri, S., Justo, D., Jauffret, C., Pavlicek, B., Dauta, A., Delmaire, C., Czernecki, V., Karachi, C., Capelle, L., Durr, A., Pessiglione, M., 2012. Critical Roles for Anterior Insula and Dorsal Striatum in Punishment-Based Avoidance Learning. Neuron 76, 998-1009. https://doi.org/10.1016/j.neuron.2012.10.017

Rolls, E.T., 2004. The functions of the orbitofrontal cortex. Brain Cogn. 55, 11-29. https://doi.org/10.1016/S0278-2626(03)00277-X

Samanez-Larkin, G.R., Hollon, N.G., Carstensen, L.L., Knutson, B., 2008. Individual differences in insular sensitivity during loss: Anticipation predict avoidance learning: Research report. Psychol. Sci. 19, 320-323. https://doi.org/10.1111/j.1467-9280.2008.02087.x

Xu, A., Larsen, B., Baller, E.B., Scott, J.C., Sharma, V., Adebimpe, A., Basbaum, A.I., Dworkin, R.H., Edwards, R.R., Woolf, C.J., Eickhoff, S.B., Eickhoff, C.R., Satterthwaite, T.D., 2020. Convergent neural representations of experimentally-induced acute pain in healthy volunteers: A large-scale fMRI meta-analysis. Neurosci. Biobehav. Rev. 112, 300-323.
