# Supplementary material for: The Neural Correlates of Individual Differences in Reinforcement Learning during Pain Avoidance and Reward Seeking
Source: eNeuro. 2024 Feb 23;11(2):ENEURO.0437-23.2024. doi: 10.1523/ENEURO.0437-23.2024 (PMC10901196; doi:10.1523/ENEURO.0437-23.2024)
Supplement: Data 1 — Code for the construction and evaluation of all RL models as described in the Methods. Fourteen models were constructed, producing 14 sets of parameters and iBIC. The optimal model included four different learning rates (εWP, εWN, εAP, and εAN) and two subjective impact of outcomes (ρW and <A), action bias b, and the Pavlovian factor π. Abbreviations: Ep: Learning rate, Rh: subjective impact of outcomes, Bi: Action bias, Pav: Pavlovian factor. Model inputs require behavioral data obtained from PLGT performance. Download Data 1, DOCX file. [file eneuro-11-ENEURO.0437-23.2024-s001.docx]

**Mediation analysis code:**

rm(list = ls())

cat("\014")

library(lavaan)

library(readxl)

setwd("/home/directory/")

mydata <- read_excel("data.xlsx")

X = mydata$brain_activity

M = mydata$learning_param

Y = mydata$behavioral

C1 = mydata$Age # covariate

C2 = mydata$Sex

C3 = mydata$Total_shocks

Data <- data.frame(X = X, Y = Y, M = M, C1 = C1, C2 = C2, C3 = C3)

mod1 <- "# a path

M ~ a * X + C1 + C2 + C3

# b path

Y ~ b * M

# c prime path

Y ~ cp * X + C1 + C2 + C3

# indirect and total effects

ab := a * b

total := cp + ab"

set.seed(1234)

fsem1 <- sem(mod1, data = Data) # , se = "bootstrap", bootstrap = 10000

summary(fsem1, standardized = TRUE)

parameterestimates(fsem1, boot.ci.type = "bca.simple", standardized = TRUE)

**Reinforcement learning model code:**

# clear

rm(list=ls())

# install Rsolnp package if you do not have it

# install.packages("Rsolnp")

library(Rsolnp)

library(MASS)# MASS is a default package for R and includes the "ginv" function for computing inverse matrix.

# import the data

subj_version = '82'

setwd("/home/directory")#please set the working directory where the data file is located.

mainfile = paste("stim_choice_return_learning_",subj_version,".csv",sep = "")

data <- read.csv(mainfile ,header = T)

savepathfig <- paste0("/output/",paste(subj_version,"_learning/",sep = ""))

dir.create(savepathfig, recursive = TRUE)

savepathgoprob <- paste0("/output/goprob_",paste(subj_version,"_learning/",sep = ""))

dir.create(savepathgoprob, recursive = TRUE)

savepathQ <- paste0("/output/Q_val_",paste(subj_version,"_learning/",sep = ""))

dir.create(savepathQ, recursive = TRUE)

savepathlearningrates <- paste0("/output/learning_rates_",paste(subj_version,"_learning/",sep = ""))

dir.create(savepathlearningrates)

savepathPE <- paste0("/output/PE_",paste(subj_version,"_learning/",sep = ""))

dir.create(savepathPE, recursive = TRUE)

#saveworkspace = paste(subj_version,"_learning_Ep4Rh2BiPav.RData",sep = "")

n <- length(unique(data$Subject))

trial <- numeric(n)

choice_all<- matrix(0, nrow = n, ncol = max(data$trial))

result_all <- choice_all

stimuli_all <- choice_all

for(i in 1:n){

trial[i] <- max(data$trial[data$Subject == i])

choice_all[i,1:trial[i]] <- data$choice[data$Subject == i]

result_all[i,1:trial[i]] <- data$return[data$Subject == i]

stimuli_all[i,1:trial[i]] <- data$stimulus[data$Subject == i]

}

# exclude the data

# ex <- c(2,48,52)

# choice_all <- choice_all[-ex,]

# result_all <- result_all[-ex,]

# stimuli_all <- stimuli_all[-ex,]

# n <- n-length(ex)

#PP <- PP[-ex]

#SP <- SP[-ex]

#Anx <- Anx[-ex]

LL <- matrix(0,nrow = n,ncol = 50)

Epsilon_RP <- LL# RP = Reward-Positive prediction error

Epsilon_RN <- LL# RN = Reward-Negative prediction error

Epsilon_PP <- LL# PP = Punishment-Positive prediction error

Epsilon_PN <- LL# PN = Punishment-Negative prediction error

Rho_R <- LL#reward

Rho_P <- LL#Punishment

Bias <- LL

Pavlov <- LL

hesseERP <- LL

hesseERN <- LL

hesseEPP <- LL

hesseEPN <- LL

hesseRR <- LL

hesseRP <- LL

hesseB <- LL

hesseP <- LL

sampling <- matrix(0,nrow = n,ncol=1000)

E4R2PB_probability <- numeric(n)

n_param <- 8# the number of free parameters in this model

s <- 8# the number of stimuli(i.e., images)

f_learn = function(param,choice,result,stimuli,trial,s){

epsilon_RP <- 1/(1+exp(-param[1]))

epsilon_RN <- 1/(1+exp(-param[2]))

epsilon_PP <- 1/(1+exp(-param[3]))

epsilon_PN <- 1/(1+exp(-param[4]))

rho_R <- exp(param[5])

rho_P <- exp(param[6])

b <- param[7]

pav <-exp(param[8])

choice <- choice

result <- result

stimuli <- stimuli

st <- result > 0 # return a boolean vector that represents the trial number where the reward is appeared.

trial <- trial

Pgo <- numeric(trial)

PE <- numeric(trial)

ll <- 0

Q <- matrix(0, nrow = s*2, ncol = trial)

V <- matrix(0, nrow = s, ncol = trial)

for (t in 1:trial){

Pgo[t] <- exp(Q[stimuli[t]+s,t]+pav*V[stimuli[t],t]+b)/(exp(Q[stimuli[t]+s,t]+pav*V[stimuli[t],t]+b) + exp(Q[stimuli[t],t]))

ll <- ll + (choice[t] == s) * log(Pgo[t]) + (choice[t] == 0) * log(1-Pgo[t])

if(any(st[stimuli == stimuli[t]])){PE[t] <- result[t] - Q[stimuli[t]+choice[t],t]/rho_R}

else{PE[t] <- result[t] - Q[stimuli[t]+choice[t],t]/rho_P}

if (t < trial){

Q[,t+1] <- Q[,t]

V[,t+1] <- V[,t]

if(any(st[stimuli == stimuli[t]])){#return true if the reward on a stimulus at trial t is appeared somewhere.

delta <- rho_R * result[t] - Q[stimuli[t]+choice[t],t]

if(delta > 0){

Q[stimuli[t]+choice[t],t+1] <- Q[stimuli[t]+choice[t],t] + epsilon_RP * delta

V[stimuli[t],t+1] <- V[stimuli[t],t] + epsilon_RP * (rho_R * result[t] - V[stimuli[t],t])

}

else{

Q[stimuli[t]+choice[t],t+1] <- Q[stimuli[t]+choice[t],t] + epsilon_RN * delta

V[stimuli[t],t+1] <- V[stimuli[t],t] + epsilon_RN * (rho_R * result[t] - V[stimuli[t],t])

}

}

else{

delta <- rho_P * result[t] - Q[stimuli[t]+choice[t],t]

if(delta > 0){

Q[stimuli[t]+choice[t],t+1] <- Q[stimuli[t]+choice[t],t] + epsilon_PP * delta

V[stimuli[t],t+1] <- V[stimuli[t],t] + epsilon_PP * (rho_P * result[t] - V[stimuli[t],t])

}

else{

Q[stimuli[t]+choice[t],t+1] <- Q[stimuli[t]+choice[t],t] + epsilon_PN * delta

V[stimuli[t],t+1] <- V[stimuli[t],t] + epsilon_PN * (rho_P * result[t] - V[stimuli[t],t])

}

}

}

}

return(list(negll = -ll, Q = Q, Pgo = Pgo, PE = PE))

}

f_minimize = function(param,choice,result,stimuli,trial,s){

results = f_learn(param,choice,result,stimuli,trial,s)

return(results$negll)

}

for (idx in 1:n){

fvalmin = Inf;

for(i in 1:10){

initial_param <- runif(n_param,0,1.0)

analyze <- solnp(initial_param,f_minimize,UB=c(20,20,20,20,3,3,Inf,3),choice = choice_all[idx,], result = result_all[idx,], stimuli = stimuli_all[idx,],trial = trial[idx], s = s)

if (analyze$values[analyze$outer.iter+1] < fvalmin){

LL[idx,1] <- fvalmin <- analyze$values[analyze$outer.iter+1]

Epsilon_RP[idx,1] <- analyze$pars[1]

Epsilon_RN[idx,1] <- analyze$pars[2]

Epsilon_PP[idx,1] <- analyze$pars[3]

Epsilon_PN[idx,1] <- analyze$pars[4]

Rho_R[idx,1] <- analyze$pars[5]

Rho_P[idx,1] <- analyze$pars[6]

Bias[idx,1] <- analyze$pars[7]

Pavlov[idx,1] <- analyze$pars[8]

#to calculate the variance of the parameter distribution, we extract the diagonal component of the inverse matrix of hessian

hesseERP[idx,1] <- diag(ginv(analyze$hessian))[1]

hesseERN[idx,1] <- diag(ginv(analyze$hessian))[2]

hesseEPP[idx,1] <- diag(ginv(analyze$hessian))[3]

hesseEPN[idx,1] <- diag(ginv(analyze$hessian))[4]

hesseRR[idx,1] <- diag(ginv(analyze$hessian))[5]

hesseRP[idx,1] <- diag(ginv(analyze$hessian))[6]

hesseB[idx,1] <- diag(ginv(analyze$hessian))[7]

hesseP[idx,1] <- diag(ginv(analyze$hessian))[8]

}

}

}

# maximum likelihood estimates

EPSILON_RP <- 1/(1+exp(-Epsilon_RP[,1]))

EPSILON_RN <- 1/(1+exp(-Epsilon_RN[,1]))

EPSILON_PP <- 1/(1+exp(-Epsilon_PP[,1]))

EPSILON_PN <- 1/(1+exp(-Epsilon_PN[,1]))

RHO_R <- exp(Rho_R[,1])

RHO_P <- exp(Rho_P[,1])

BIAS <- Bias[,1]

PAVLOV <- exp(Pavlov[,1])

LL_E4R2PB <- LL[,1]#sum(2*LL_E4R2PB + n_param*log(sum(trial)))

f_plot = function(id,im,x = 1){#id = participants'ID; im = image, x = set the parameters in x th iteration in the EM-algorithm (x is set to 1 by default).

prob <- f_learn(c(Epsilon_RP[id,x],Epsilon_RN[id,x],Epsilon_PP[id,x],Epsilon_PN[id,x],Rho_R[id,x],Rho_P[id,x],Bias[id,x],Pavlov[id,x]),choice_all[id,],result_all[id,],stimuli_all[id,],trial[id],s)$Pgo

c_I <- choice_all[id,1:trial[id]][stimuli_all[id,1:trial[id]] == im]/s

r_I <- 1+abs(result_all[id,1:trial[id]][stimuli_all[id,1:trial[id]] == im])

t_I <- 1:sum(stimuli_all[id,1:trial[id]]==im)

plot(t_I,prob[stimuli_all[id,1:trial[id]]==im], type = "l",ylim = c(0.0,1.1),xlab = "trial",ylab = "p(go)")

par(new =T)

plot(t_I,c_I,ylim = c(0.0,1.1),col = r_I,ann = F)

}

#show the plot of the data for the ID number 1 in image 1

f_plot(1,1)

#The line indicates the probabilities the model predicted.

#The circles indicate participants'choices: 1 = go, 0 = no-go.

#The color of the circle indicates whether the outcome appeared: red = present, black = absent.

m <- length(unique(data$stimulus))

for(i1 in 1:n){

for(j1 in 1:m){

f_plot(i1,j1) #show the plot of the data for the ID number i in image j

# filename = paste0(mypath,paste("subject",i,"_image",j,".pdf",sep = ""))

# dev.print(pdf, filename)

# subject <- data$ID == i

subjectid <- data$ID[data$Subject == i1]

filename1 = paste0(savepathfig,paste(subjectid[1],"_image",j1,".jpg",sep = ""))

dev.copy(jpeg,filename1, units="in", width=5, height=5, res=300);

dev.off ();

}

}

l <- 20#this is a number of trials to plot mean Q values.

tmp <- matrix(0,nrow = m, ncol = l)

q_gw <- matrix(0,nrow = n, ncol = l)

q_ngw <- q_gw

q_ga <- q_gw

q_nga <- q_gw

# save go probabilities and Q value

for(i2 in 1:n){

for(j2 in 1:m){

id = i2

x = 1

im = j2

prob <- f_learn(c(Epsilon_RP[id,x],Epsilon_RN[id,x],Epsilon_PP[id,x],Epsilon_PN[id,x],Rho_R[id,x],Rho_P[id,x],Bias[id,x],Pavlov[id,x]),choice_all[id,],result_all[id,],stimuli_all[id,],trial[id],s)$Pgo

Q_val <- f_learn(c(Epsilon_RP[id,x],Epsilon_RN[id,x],Epsilon_PP[id,x],Epsilon_PN[id,x],Rho_R[id,x],Rho_P[id,x],Bias[id,x],Pavlov[id,x]),choice_all[id,],result_all[id,],stimuli_all[id,],trial[id],s)$Q

stimprob <- prob[stimuli_all[id,1:trial[id]]==im] # extract each stimulus separately

if(im < 5){

stimQ_nogo <- Q_val[im,][stimuli_all[id,1:trial[id]]==im]/exp(Rho_R[id,x])#

stimQ_go <- Q_val[im+m,][stimuli_all[id,1:trial[id]]==im]/exp(Rho_R[id,x])#

}

else{

stimQ_nogo <- Q_val[im,][stimuli_all[id,1:trial[id]]==im]/exp(Rho_P[id,x])#

stimQ_go <- Q_val[im+m,][stimuli_all[id,1:trial[id]]==im]/exp(Rho_P[id,x])#

}

subjectid <- data$ID[data$Subject == i2]

filename2a = paste0(savepathgoprob,paste(subjectid[1],"_image",j2,".csv",sep = ""))

filename2b = paste0(savepathQ,paste(subjectid[1],"_image",j2,"_no-go.csv",sep = ""))

filename2c = paste0(savepathQ,paste(subjectid[1],"_image",j2,"_go.csv",sep = ""))

savedata1a <- data.frame(stimprob) # save go prob for each stimulus

savedata1b <- data.frame(stimQ_nogo)# save Q value of no-go action for each stimulus

savedata1c <- data.frame(stimQ_go)# save Q value go action for each stimulus

write.csv(savedata1a, filename2a)

write.csv(savedata1b, filename2b)

write.csv(savedata1c, filename2c)

filename3a = paste0(savepathgoprob,paste(subjectid[1],"_all.csv",sep = ""))

filename3b = paste0(savepathQ,paste(subjectid[1],"_all.csv",sep = ""))

savedata2a <- data.frame(prob) # save go prob for all trials

savedata2b <- data.frame(Q_val)# save Q value for all trials

write.csv(savedata2a, filename3a)

write.csv(savedata2b, filename3b)

tmp[im,] <- stimQ_go[1:l] - stimQ_nogo[1:l]

}

q_gw[id,] <- colMeans(tmp[1:2,])

q_ngw[id,] <- colMeans(tmp[3:4,])

q_ga[id,] <- colMeans(tmp[5:6,])

q_nga[id,] <- colMeans(tmp[7:8,])

}

for(i3 in 1:n){

for(j3 in 1:m){

id = i3

x = 1

im = j3

PE_val <- f_learn(c(Epsilon_RP[id,x],Epsilon_RN[id,x],Epsilon_PP[id,x],Epsilon_PN[id,x],Rho_R[id,x],Rho_P[id,x],Bias[id,x],Pavlov[id,x]),choice_all[id,],result_all[id,],stimuli_all[id,],trial[id],s)$PE

#Please use this function for extracting the PE. The PE is output in the same format as the Pgo values.

stimPE <- PE_val[stimuli_all[id,1:trial[id]]==im] # extract each stimulus separately

subjectid <- data$ID[data$Subject == i3]

filenamePE = paste0(savepathPE,paste(subjectid[1],"_image",j3,".csv",sep = ""))

savedataPE <- data.frame(stimPE) # save PE for each stimulus

write.csv(savedataPE, filenamePE)

filenamePE_all = paste0(savepathPE,paste(subjectid[1],"_all.csv",sep = ""))

savedataPE_all <- data.frame(PE_val) # save PE for all stimuli

write.csv(savedataPE_all, filenamePE_all)

}

}

# estimate hyper parameters of the posterior distribitions

EpsilonRP_mu <- mean(Epsilon_RP[,1])

EpsilonRP_sigma <- sum(Epsilon_RP[,1]^2 + hesseERP[,1])/n - EpsilonRP_mu^2

EpsilonRN_mu <- mean(Epsilon_RN[,1])

EpsilonRN_sigma <- sum(Epsilon_RN[,1]^2 + hesseERN[,1])/n - EpsilonRN_mu^2

EpsilonPP_mu <- mean(Epsilon_PP[,1])

EpsilonPP_sigma <- sum(Epsilon_PP[,1]^2 + hesseEPP[,1])/n - EpsilonPP_mu^2

EpsilonPN_mu <- mean(Epsilon_PN[,1])

EpsilonPN_sigma <- sum(Epsilon_PN[,1]^2 + hesseEPN[,1])/n - EpsilonPN_mu^2

RhoR_mu <- mean(Rho_R[,1])

RhoR_sigma <- sum(Rho_R[,1]^2 + hesseRR[,1])/n - RhoR_mu^2

RhoP_mu <- mean(Rho_P[,1])

RhoP_sigma <- sum(Rho_P[,1]^2 + hesseRP[,1])/n - RhoP_mu^2

Bias_mu <- mean(Bias[,1])

Bias_sigma <- sum(Bias[,1]^2 + hesseB[,1])/n - Bias_mu^2

Pavlov_mu <- mean(Pavlov[,1])

Pavlov_sigma <- sum(Pavlov[,1]^2 + hesseP[,1])/n - Pavlov_mu^2

# calculate the convergence criterion

QERP <- -(n*log(2*pi*EpsilonRP_sigma))/2-sum((Epsilon_RP[,1]^2 + hesseERP[,1] - 2*Epsilon_RP[,1]*EpsilonRP_mu + EpsilonRP_mu^2)/(2*EpsilonRP_sigma))

QERN <- -(n*log(2*pi*EpsilonRN_sigma))/2-sum((Epsilon_RN[,1]^2 + hesseERN[,1] - 2*Epsilon_RN[,1]*EpsilonRN_mu + EpsilonRN_mu^2)/(2*EpsilonRN_sigma))

QEPP <- -(n*log(2*pi*EpsilonPP_sigma))/2-sum((Epsilon_PP[,1]^2 + hesseEPP[,1] - 2*Epsilon_PP[,1]*EpsilonPP_mu + EpsilonPP_mu^2)/(2*EpsilonPP_sigma))

QEPN <- -(n*log(2*pi*EpsilonPN_sigma))/2-sum((Epsilon_PN[,1]^2 + hesseEPN[,1] - 2*Epsilon_PN[,1]*EpsilonPN_mu + EpsilonPN_mu^2)/(2*EpsilonPN_sigma))

QRR <- -(n*log(2*pi*RhoR_sigma))/2-sum((Rho_R[,1]^2 + hesseRR[,1] - 2*Rho_R[,1]*RhoR_mu + RhoR_mu^2)/(2*RhoR_sigma))

QRP <- -(n*log(2*pi*RhoP_sigma))/2-sum((Rho_P[,1]^2 + hesseRP[,1] - 2*Rho_P[,1]*RhoP_mu + RhoP_mu^2)/(2*RhoP_sigma))

QB <- -(n*log(2*pi*Bias_sigma))/2-sum((Bias[,1]^2 + hesseB[,1] - 2*Bias[,1]*Bias_mu + Bias_mu^2)/(2*Bias_sigma))

QP <- -(n*log(2*pi*Pavlov_sigma))/2-sum((Pavlov[,1]^2 + hesseP[,1] - 2*Pavlov[,1]*Pavlov_mu + Pavlov_mu^2)/(2*Pavlov_sigma))

Q_value <- numeric(50)

Q_value[1] <- QERP + QERN + QEPP + QEPN + QRR + QRP + QB + QP

f_Q = function(param, choice, result, stimuli, trial, s){

epsilon_RP <- 1/(1+exp(-param[1]))

epsilon_RN <- 1/(1+exp(-param[2]))

epsilon_PP <- 1/(1+exp(-param[3]))

epsilon_PN <- 1/(1+exp(-param[4]))

rho_R <- exp(param[5])

rho_P <- exp(param[6])

b <- param[7]

pav <-exp(param[8])

choice <- choice

result <- result

stimuli <- stimuli

st <- result > 0 # return a boolean vector that represents the trial number where the reward is appeared.

trial <- trial

Pgo <- numeric(trial)

ll <- 0

Q <- matrix(0, nrow = s*2, ncol = trial)

V <- matrix(0, nrow = s, ncol = trial)

for (t in 1:trial){

Pgo[t] <- exp(Q[stimuli[t]+s,t]+pav*V[stimuli[t],t]+b)/(exp(Q[stimuli[t]+s,t]+pav*V[stimuli[t],t]+b) + exp(Q[stimuli[t],t]))

ll <- ll + (choice[t] == s) * log(Pgo[t]) + (choice[t] == 0) * log(1-Pgo[t])

if (t < trial){

Q[,t+1] <- Q[,t]

V[,t+1] <- V[,t]

if(any(st[stimuli == stimuli[t]])){#return true if the reward on a stimulus at trial t is appeared somewhere.

delta <- rho_R * result[t] - Q[stimuli[t]+choice[t],t+1]

if(delta > 0){

Q[stimuli[t]+choice[t],t+1] <- Q[stimuli[t]+choice[t],t] + epsilon_RP * delta

V[stimuli[t],t+1] <- V[stimuli[t],t] + epsilon_RP * (rho_R * result[t] - V[stimuli[t],t])

}

else{

Q[stimuli[t]+choice[t],t+1] <- Q[stimuli[t]+choice[t],t] + epsilon_RN * delta

V[stimuli[t],t+1] <- V[stimuli[t],t] + epsilon_RN * (rho_R * result[t] - V[stimuli[t],t])

}

}

else{

delta <- rho_P * result[t] - Q[stimuli[t]+choice[t],t+1]

if(delta > 0){

Q[stimuli[t]+choice[t],t+1] <- Q[stimuli[t]+choice[t],t] + epsilon_PP * delta

V[stimuli[t],t+1] <- V[stimuli[t],t] + epsilon_PP * (rho_P * result[t] - V[stimuli[t],t])

}

else{

Q[stimuli[t]+choice[t],t+1] <- Q[stimuli[t]+choice[t],t] + epsilon_PN * delta

V[stimuli[t],t+1] <- V[stimuli[t],t] + epsilon_PN * (rho_P * result[t] - V[stimuli[t],t])

}

}

}

}

ll <- ll+ log(dnorm(param[1],EpsilonRP_mu,sqrt(EpsilonRP_sigma))) + log(dnorm(param[2],EpsilonRN_mu,sqrt(EpsilonRN_sigma))) + log(dnorm(param[3],EpsilonPP_mu,sqrt(EpsilonPP_sigma))) + log(dnorm(param[4],EpsilonPN_mu,sqrt(EpsilonPN_sigma)))+log(dnorm(param[5],RhoR_mu,sqrt(RhoR_sigma)))+log(dnorm(param[6],RhoP_mu,sqrt(RhoP_sigma)))+log(dnorm(param[7],Bias_mu,sqrt(Bias_sigma)))+log(dnorm(param[8],Pavlov_mu,sqrt(Pavlov_sigma)))

return(negll = -ll)

}

for(x in 2:length(Q_value)){

for (idx in 1:n){

fvalmin = Inf;

for(i in 1:10){

initial_param <- runif(n_param,0,1.0)

analyze <- solnp(initial_param,f_Q,UB=c(20,20,20,20,3,3,Inf,3),choice = choice_all[idx,], result = result_all[idx,], stimuli = stimuli_all[idx,], trial = trial[idx], s = s)#

if (analyze$values[analyze$outer.iter+1] < fvalmin){

LL[idx,x] <- fvalmin <- analyze$values[analyze$outer.iter+1]

Epsilon_RP[idx,x] <- analyze$pars[1]

Epsilon_RN[idx,x] <- analyze$pars[2]

Epsilon_PP[idx,x] <- analyze$pars[3]

Epsilon_PN[idx,x] <- analyze$pars[4]

Rho_R[idx,x] <- analyze$pars[5]

Rho_P[idx,x] <- analyze$pars[6]

Bias[idx,x] <- analyze$pars[7]

Pavlov[idx,x] <- analyze$pars[8]

#to calculate the variance of the parameter distribution, we extract the diagonal component of the inverse matrix of hessian

hesseERP[idx,x] <- diag(ginv(analyze$hessian))[1]

hesseERN[idx,x] <- diag(ginv(analyze$hessian))[2]

hesseEPP[idx,x] <- diag(ginv(analyze$hessian))[3]

hesseEPN[idx,x] <- diag(ginv(analyze$hessian))[4]

hesseRR[idx,x] <- diag(ginv(analyze$hessian))[5]

hesseRP[idx,x] <- diag(ginv(analyze$hessian))[6]

hesseB[idx,x] <- diag(ginv(analyze$hessian))[7]

hesseP[idx,x] <- diag(ginv(analyze$hessian))[8]

}

}

}

EpsilonRP_mu <- mean(Epsilon_RP[,x])

EpsilonRP_sigma <- sum(Epsilon_RP[,x]^2 + hesseERP[,x])/n - EpsilonRP_mu^2

EpsilonRN_mu <- mean(Epsilon_RN[,x])

EpsilonRN_sigma <- sum(Epsilon_RN[,x]^2 + hesseERN[,x])/n - EpsilonRN_mu^2

EpsilonPP_mu <- mean(Epsilon_PP[,x])

EpsilonPP_sigma <- sum(Epsilon_PP[,x]^2 + hesseEPP[,x])/n - EpsilonPP_mu^2

EpsilonPN_mu <- mean(Epsilon_PN[,x])

EpsilonPN_sigma <- sum(Epsilon_PN[,x]^2 + hesseEPN[,x])/n - EpsilonPN_mu^2

RhoR_mu <- mean(Rho_R[,x])

RhoR_sigma <- sum(Rho_R[,x]^2 + hesseRR[,x])/n - RhoR_mu^2

RhoP_mu <- mean(Rho_P[,x])

RhoP_sigma <- sum(Rho_P[,x]^2 + hesseRP[,x])/n - RhoP_mu^2

Bias_mu <- mean(Bias[,x])

Bias_sigma <- sum(Bias[,x]^2 + hesseB[,x])/n - Bias_mu^2

Pavlov_mu <- mean(Pavlov[,x])

Pavlov_sigma <- sum(Pavlov[,x]^2 + hesseP[,x])/n - Pavlov_mu^2

QERP <- -(n*log(2*pi*EpsilonRP_sigma))/2-sum((Epsilon_RP[,x]^2 + hesseERP[,x] - 2*Epsilon_RP[,x]*EpsilonRP_mu + EpsilonRP_mu^2)/(2*EpsilonRP_sigma))

QERN <- -(n*log(2*pi*EpsilonRN_sigma))/2-sum((Epsilon_RN[,x]^2 + hesseERN[,x] - 2*Epsilon_RN[,x]*EpsilonRN_mu + EpsilonRN_mu^2)/(2*EpsilonRN_sigma))

QEPP <- -(n*log(2*pi*EpsilonPP_sigma))/2-sum((Epsilon_PP[,x]^2 + hesseEPP[,x] - 2*Epsilon_PP[,x]*EpsilonPP_mu + EpsilonPP_mu^2)/(2*EpsilonPP_sigma))

QEPN <- -(n*log(2*pi*EpsilonPN_sigma))/2-sum((Epsilon_PN[,x]^2 + hesseEPN[,x] - 2*Epsilon_PN[,x]*EpsilonPN_mu + EpsilonPN_mu^2)/(2*EpsilonPN_sigma))

QRR <- -(n*log(2*pi*RhoR_sigma))/2-sum((Rho_R[,x]^2 + hesseRR[,x] - 2*Rho_R[,x]*RhoR_mu + RhoR_mu^2)/(2*RhoR_sigma))

QRP <- -(n*log(2*pi*RhoP_sigma))/2-sum((Rho_P[,x]^2 + hesseRP[,x] - 2*Rho_P[,x]*RhoP_mu + RhoP_mu^2)/(2*RhoP_sigma))

QB <- -(n*log(2*pi*Bias_sigma))/2-sum((Bias[,x]^2 + hesseB[,x] - 2*Bias[,x]*Bias_mu + Bias_mu^2)/(2*Bias_sigma))

QP <- -(n*log(2*pi*Pavlov_sigma))/2-sum((Pavlov[,x]^2 + hesseP[,x] - 2*Pavlov[,x]*Pavlov_mu + Pavlov_mu^2)/(2*Pavlov_sigma))

Q_value[x] <- QERP + QERN + QEPP + QEPN + QRR + QRP + QB + QP

if(abs(Q_value[x] - Q_value[x-1]) < 0.01) break

}

Ep_RP <- 1/(1+exp(-Epsilon_RP[,x]))

Ep_RN <- 1/(1+exp(-Epsilon_RN[,x]))

Ep_PP <- 1/(1+exp(-Epsilon_PP[,x]))

Ep_PN <- 1/(1+exp(-Epsilon_PN[,x]))

Rh_R <-exp(Rho_R[,x])

Rh_P <-exp(Rho_P[,x])

Bi <- Bias[,x]

Pav <-exp(Pavlov[,x])

# calculate iBIC value

for (idx in 1:n){

for (k in 1:1000){

epsilonRP_k <- rnorm(1,EpsilonRP_mu,sqrt(EpsilonRP_sigma))

epsilonRN_k <- rnorm(1,EpsilonRN_mu,sqrt(EpsilonRN_sigma))

epsilonPP_k <- rnorm(1,EpsilonPP_mu,sqrt(EpsilonPP_sigma))

epsilonPN_k <- rnorm(1,EpsilonPN_mu,sqrt(EpsilonPN_sigma))

rhoR_k <- rnorm(1,RhoR_mu,sqrt(RhoR_sigma))

rhoP_k <- rnorm(1,RhoP_mu,sqrt(RhoP_sigma))

bias_k <- rnorm(1,Bias_mu,sqrt(Bias_sigma))

pav_k <- rnorm(1,Pavlov_mu,sqrt(Pavlov_sigma))

sampling[idx,k] <- f_minimize(c(epsilonRP_k,epsilonRN_k,epsilonPP_k,epsilonPN_k,rhoR_k,rhoP_k,bias_k,pav_k),choice_all[idx,],result_all[idx,],stimuli_all[idx,],trial[idx],s)

if(is.nan(sampling[idx,k])){

while(is.nan(sampling[idx,k])){

epsilonRP_k <- rnorm(1,EpsilonRP_mu,sqrt(EpsilonRP_sigma))

epsilonRN_k <- rnorm(1,EpsilonRN_mu,sqrt(EpsilonRN_sigma))

epsilonPP_k <- rnorm(1,EpsilonPP_mu,sqrt(EpsilonPP_sigma))

epsilonPN_k <- rnorm(1,EpsilonPN_mu,sqrt(EpsilonPN_sigma))

rhoR_k <- rnorm(1,RhoR_mu,sqrt(RhoR_sigma))

rhoP_k <- rnorm(1,RhoP_mu,sqrt(RhoP_sigma))

bias_k <- rnorm(1,Bias_mu,sqrt(Bias_sigma))

pav_k <- rnorm(1,Pavlov_mu,sqrt(Pavlov_sigma))

sampling[idx,k] <- f_minimize(c(epsilonRP_k,epsilonRN_k,epsilonPP_k,epsilonPN_k,rhoR_k,rhoP_k,bias_k,pav_k),choice_all[idx,],result_all[idx,],stimuli_all[idx,],trial[idx],s)

}

}

if(is.infinite(sampling[idx,k])){

epsilonRP_k <- rnorm(1,EpsilonRP_mu,sqrt(EpsilonRP_sigma))

epsilonRN_k <- rnorm(1,EpsilonRN_mu,sqrt(EpsilonRN_sigma))

epsilonPP_k <- rnorm(1,EpsilonPP_mu,sqrt(EpsilonPP_sigma))

epsilonPN_k <- rnorm(1,EpsilonPN_mu,sqrt(EpsilonPN_sigma))

rhoR_k <- rnorm(1,RhoR_mu,sqrt(RhoR_sigma))

rhoP_k <- rnorm(1,RhoP_mu,sqrt(RhoP_sigma))

bias_k <- rnorm(1,Bias_mu,sqrt(Bias_sigma))

pav_k <- rnorm(1,Pavlov_mu,sqrt(Pavlov_sigma))

sampling[idx,k] <- f_minimize(c(epsilonRP_k,epsilonRN_k,epsilonPP_k,epsilonPN_k,rhoR_k,rhoP_k,bias_k,pav_k),choice_all[idx,],result_all[idx,],stimuli_all[idx,],trial[idx],s)

}

}

E4R2PB_probability[idx] <- log(sum(exp(-sampling[idx,]))/1000)#log(mean(exp(-sampling[idx,])))

}

# The penalty term for the number of free parameter is twice the number of free parameters, because we used the mean and variance of each free parameter

iBIC_E4R2PB <- -2*sum(E4R2PB_probability)+n_param*2*log(sum(trial))

saveworkspace = paste(subj_version,"_Ep4Rh2BiPav_learning.RData",sep = "")

save.image(file = saveworkspace) # save all workspace

# save model outputs

filename4 = paste0(savepathlearningrates,paste("learning_rates.csv",sep = ""))

IDs = unique(data$ID)

savedata_params <- data.frame(IDs,EPSILON_RP,EPSILON_RN,EPSILON_PP,EPSILON_PN,Ep_RP,Ep_RN,Ep_PP,Ep_PN)

write.csv(savedata_params, filename4) # , row.names=FALSE
